# SAFPred: synteny-aware gene function prediction for bacteria using protein embeddings

**DOI:** 10.1093/bioinformatics/btae328

**Published:** 2024-05-22

**Authors:** Aysun Urhan, Bianca-Maria Cosma, Ashlee M Earl, Abigail L Manson, Thomas Abeel

**Affiliations:** Delft Bioinformatics Lab, Delft University of Technology Van Mourik, Delft XE 2628, The Netherlands; Infectious Disease and Microbiome Program, Broad Institute of MIT and Harvard, Cambridge, MA 02142, United States; Delft Bioinformatics Lab, Delft University of Technology Van Mourik, Delft XE 2628, The Netherlands; Infectious Disease and Microbiome Program, Broad Institute of MIT and Harvard, Cambridge, MA 02142, United States; Infectious Disease and Microbiome Program, Broad Institute of MIT and Harvard, Cambridge, MA 02142, United States; Delft Bioinformatics Lab, Delft University of Technology Van Mourik, Delft XE 2628, The Netherlands; Infectious Disease and Microbiome Program, Broad Institute of MIT and Harvard, Cambridge, MA 02142, United States

## Abstract

**Motivation:**

Today, we know the function of only a small fraction of the protein sequences predicted from genomic data. This problem is even more salient for bacteria, which represent some of the most phylogenetically and metabolically diverse taxa on Earth. This low rate of bacterial gene annotation is compounded by the fact that most function prediction algorithms have focused on eukaryotes, and conventional annotation approaches rely on the presence of similar sequences in existing databases. However, often there are no such sequences for novel bacterial proteins. Thus, we need improved gene function prediction methods tailored for bacteria. Recently, transformer-based language models—adopted from the natural language processing field—have been used to obtain new representations of proteins, to replace amino acid sequences. These representations, referred to as protein embeddings, have shown promise for improving annotation of eukaryotes, but there have been only limited applications on bacterial genomes.

**Results:**

To predict gene functions in bacteria, we developed SAFPred, a novel synteny-aware gene function prediction tool based on protein embeddings from state-of-the-art protein language models. SAFpred also leverages the unique operon structure of bacteria through conserved synteny. SAFPred outperformed both conventional sequence-based annotation methods and state-of-the-art methods on multiple bacterial species, including for distant homolog detection, where the sequence similarity to the proteins in the training set was as low as 40%. Using SAFPred to identify gene functions across diverse enterococci, of which some species are major clinical threats, we identified 11 previously unrecognized putative novel toxins, with potential significance to human and animal health.

**Availability and implementation:**

https://github.com/AbeelLab/safpred.

## 1 Introduction

With increasing volumes of sequencing data from high-throughput technologies, the observed diversity of protein sequences is increasing faster than our knowledge of its function. Given costs and the inability to scale experimental and other manual approaches for function prediction, computational approaches have a critical role in deciphering functional diversity. Most state-of-the-art gene function prediction methods have focused on eukaryotes, leaving a gap in our understanding of the vast landscape of diversity among bacteria, which represent some of the most phylogenetically and metabolically diverse taxa.

As with previous tools, we define gene function prediction as the process of mapping terms from the Gene Ontology (GO) knowledgebase to ORFs where the start and stop positions have been annotated (Ashburner *et al.* 2000, Zhou *et al.* 2019). Conventional approaches for gene function prediction rely on sequence homology. Initial methods employed sequence search tools such as BLAST or DIAMOND to query a database of known protein sequences and their functions (Altschul *et al.* 1990). While useful, these methods are limited by the completeness and fidelity of their databases. Furthermore, it is often difficult to determine appropriate thresholds, resulting in low sensitivity and specificity (Zhou *et al.* 2019). With increasing data, machine learning techniques have been explored; in the most recent Critical Assessment of Functional Annotation (CAFA), a challenge established to evaluate the state-of-the-art in automated function prediction, GOLabeler was the top performer for predicting molecular function ontologies by integrating sequence alignments, domain and motif information, and biophysical properties of proteins (You *et al.* 2018).

More recently, deep learning methods leveraging ideas from natural language processing (NLP) have gained attention. Deep learning-based protein language models were recently used to extract embedding vectors for protein sequences that are analogous to word embeddings (Heinzinger *et al.* 2019, Elnaggar *et al.* 2020, Rives *et al.* 2021). These vectors capture core properties of proteins beyond primary structure, in a way that is context and species agnostic, but relevant to their function in the cell, which makes them particularly useful for understudied organisms (Hoarfrost *et al.* 2022). Contextualized word embeddings have demonstrated success in predicting GO terms, as well as structure and localization prediction, and refining protein family clusters (Littmann *et al.* 2021).

Compared to eukaryotes, much less has been done to apply NLP-based methods to bacterial genes. In a recent CAFA challenge, methods consistently performed less well on bacteria than eukaryotes, suggesting room for improvement. Furthermore, the prokaryotic track was heavily biased toward a single, well-studied bacterial species, *E. coli* (Zhou *et al.* 2019), pointing to a need to test methodologies on diverse bacteria. However, more recently, Mahlich *et al.* (2018, 2023) showed that with more sophisticated deep-learning methods to study bacterial function, an incredible amount of knowledge can be gained about remote homologs in novel organisms. Given the vast diversity of functional repertoire in bacteria, remote homology detection is of utmost importance.

Many functionally related bacterial genes are encoded in operons, colocated clusters of genes on the same strand, which are often coregulated and cotranscribed. Thus, the context of a gene is another means to infer clues to its function (de Daruvar *et al.* 2002, Li *et al.* 2009), as it is a source of information complementary to both the sequence and embeddings-based representation of a gene. Leveraging gene context and interactions was shown to improve prediction performance on eukaryotes (Makrodimitris *et al.* 2020, Yao *et al.* 2021); however, combining information from gene context with embeddings-based gene representations has not yet been done for gene function prediction.

We developed Synteny-Aware Function Predictor (SAFPred), a novel approach to improve bacterial gene function prediction based on protein embeddings and a comprehensive bacterial synteny database. To evaluate SAFPred, we performed extensive benchmarking using ground truth data and automated function prediction standard approaches to show that SAFPred outperformed conventional sequence-based bacterial genome annotation pipelines, HMM-based approaches, and a state-of-the-art deep learning method, when using gene synteny conservation as additional input. As part of a real-world application, we also demonstrated SAFPred’s utility to predict protein functions in *Enterococcus* species, including predicting potential novel pore-forming toxins related to the delta toxin family that could not be recognized using linear sequence or protein domain information. SAFPred provides a powerful new tool for gene function prediction in bacteria, combining state-of-the-art NLP methods with a novel incorporation of syntenic information for bacteria.

## 2 Materials and methods

### 2.1 Datasets

#### 2.1.1 SwissProt dataset for benchmarking

We retrieved all the manually reviewed entries from the SwissProt Database (release 2021-04, retrieval date 10 November 2021) (The UniProt Consortium 2018), which was filtered to include proteins of length 40–1000 amino acids and with at least one experimental GO annotation. We selected the evidence codes EXP, IDA, IPI, IMP, IGI, IEP, HTP, HDA, HMP, HGI, HEP, IBA, IBD, IKR, IRD, IC, and TAS. To reduce redundancy, we clustered the proteins using CD-HIT (Li and Godzik 2006) at 95% sequence similarity. The final dataset comprised 107 818 proteins in total.

To benchmark the performance of our method, we created five benchmarking datasets from SwissProt, one for each of the five most numerous bacterial organisms in our dataset (Table 1). Each organism’s dataset was split into training and test sets. The test was set composed of all proteins from the specific bacteria. We also divided each training set in different ways to create five sets where the sequence similarity (calculated using BLASTp) (Altschul *et al.* 1990) of test to training set proteins was at most 40%, 50%, 60%, 70%, and 80%. This resulted in a total of 30 benchmarking sets (Table 1 and Supplementary Text).

**Table 1.** Total number of proteins in the benchmarking sets generated from the SwissProt dataset to evaluate function prediction tools on bacterial organisms.

Organism name	No. of proteins in the test set	No. of proteins in the training set (for given similarity between training and test sets)					
		40%	50%	60%	70%	80%	95% (Full)^a^
*Escherichia coli (EC)*	3454	87 014	96 471	100 445	102 262	103 229	104 377
*Mycobacterium tuberculosis (MT)*	1666	95 367	102 531	105 158	105 917	106 114	106 152
*Bacillus subtilis (BS)*	1636	93 363	101 112	104 325	105 609	106 015	106 182
*Pseudomonas aeruginosa (PA)*	1014	94 679	101 338	104 644	106 186	106 680	106 804
*Salmonella typhimurium (ST)*	774	100 928	104 164	105 384	105 980	106 340	107 044

For each organism, the test set remained constant and was composed of all entries from the specific bacterial species, whereas the training set was restricted according to the maximum sequence similarity allowed between the test and training sets.

a 95% similarity was chosen to represent the full dataset to avoid redundancy.

#### 2.1.2 *Enterococcus* diversity dataset

We applied SAFPred to a set of 61 746 proteins with no experimental annotations, representing the entire protein content of 19 *Enterococcus* species, spanning four *Enterococcus* clades (Lebreton *et al.* 2017) (Supplementary Table S5). This collection of genomes is representative of *Enterococcus* genomic diversity, hence we refer to it as the *Enterococcus* diversity dataset. Assemblies were downloaded from the Assembly Database in NCBI.

### 2.2 Building the bacterial synteny database, SAFPredDB

**Figure 1.**
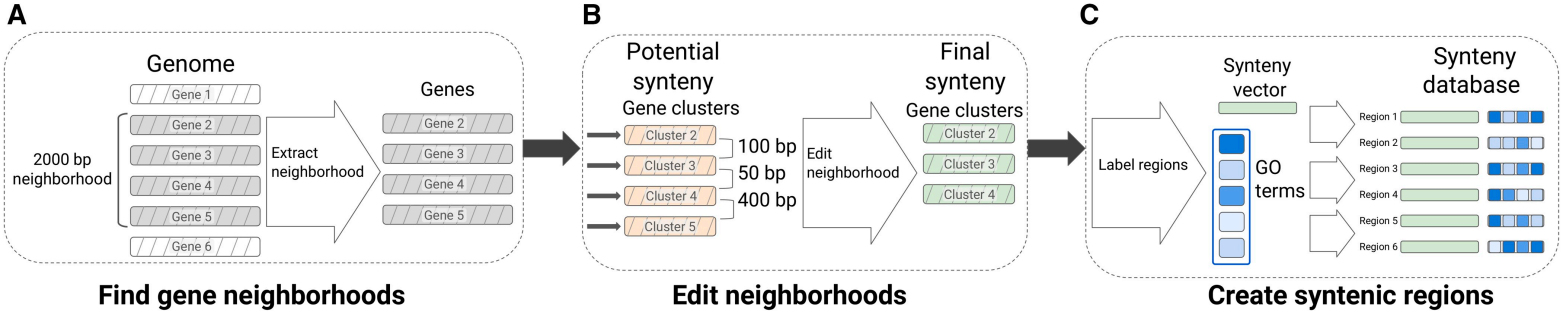
Schematic diagram of method used to construct our synteny database, SAFPredDB. Hashed boxes represent genes; solid boxes are numerical embedding vectors. (A) 2000-bp-long gene neighborhoods are extracted from all genomes in GTDB; shown is an example with four genes in a single genomic neighborhood (hashed grey boxes). (B) After clustering all proteins from GTDB with CD-HIT, we replace the genes with the CD-HIT clusters they belong to (hashed orange boxes) using the amino-acid sequence of the representative gene of each cluster in place of their actual amino-acid sequence. Then, we trim potential syntenic regions to remove genes separated by > 300 bp, resulting in final syntenic regions (hashed green boxes). (C) Once the final syntenic regions are determined, we (i) annotate each region with a set of GO terms, for which we track the corresponding frequency among the gene clusters that make up the region (blue rectangles, darker shades mean GO terms are found in more genes within the region), and (ii) extract numerical embedding vectors for each region (solid green boxes). We create a new representation for each region, which consists of the average embedding vector and a set of GO terms. The final synteny database is a collection of such representative embedding vectors and GO term frequency vectors; representations of six example entries are shown here.

SAFPredDB is a comprehensive compilation of bacterial syntenic relationships, designed as a resource for SAFPred. It is based on genomic data from the Genome Taxonomy Database (GTDB Release 202, retrieved on 31 March 2022) (Parks *et al.* 2021) because GTDB assigns representative genomes based on assembly quality and provides a curated list of species, with consistent labels and IDs to cross-reference to all other databases. Starting with 45 555 representative genomes, we extracted all protein sequences from the standardized GTDB annotations and clustered them using CD-HIT at 95% sequence identity with default parameters, keeping only clusters that contained at least 10 genes, resulting in 372 308 clusters. Next, we identified synteny by grouping clusters if at least one cluster member was located on the same contig and strand, within 2000 bp (Fig. 1A). This yielded 1 488 249 nonsingleton candidate regions. Finally, we removed regions with an intergenic distance >300 bp, or split them into multiple regions if possible (Fig. 1B). At the end of this procedure, SAFPredDB consisted of 406 293 unique nonsingleton regions, and the largest region was 25 genes long.

We used experimentally determined operons collected in the Operon DataBase (ODB v4) (Okuda and Yoshizawa 2010) to help determine threshold values used when building SAFPredDB, and to validate SAFPredDB. We downloaded both the ODB known and the ODB conserved operon databases on 31 March 2022. We identified operons in ODB belonging to *E. coli* and *B. subtilis*, as (i) these two organisms form the basis of a large part of the benchmarking of SAFPredDB, (ii) we could cross-reference the protein IDs in ODB to the locus tags in their respective genome assemblies, and (iii) they are two of the most well-represented organisms in ODB. The ODB conserved operon database contained 8235 unique operons, from which we extracted descriptive statistics and common patterns found across several operons conserved among bacterial organisms. The ODB known operon database was used to model synteny features and determine thresholds, such as region length, number of genes in a region, and the maximum intergenic distance between adjacent genes in a region.

To summarize each SAFPredDB entry, we extracted protein embedding vectors for the representative sequence of clusters found in that entry. We used ESM-1b, a transformer-based protein language model (Rives *et al.* 2021) to extract the embeddings, and we took the average of these embeddings to obtain one embedding vector per operon (Fig. 1C). Then, we annotated SAFPredDB entries by assigning GO terms, if possible. Since we did not have experimental annotations, we labeled entries based on sequence similarity. We used BLASTp (Altschul *et al.* 1990) to calculate pairwise sequence similarity between proteins in SAFPredDB entries and the nonredundant SwissProt database with experimentally determined GO terms (all 107 818 entries). We transferred GO terms from significant hits (e-value <1e-6 and bit score >50) using the frequency of each GO term among these hits as a predicted score. We could assign at least one GO term to 295 446 of the 372 308 clusters (79%), which in turn yielded 388 377 nonsingleton entries (out of 406 293; 96%) annotated with at least one GO term (Supplementary Table S2).

In order to keep our synteny database consistent with our benchmarking datasets, where we evaluated SAFPred on training subsets with differing sequence similarity to the proteins in the test set, we generated corresponding subsets of SAFPredDB with matching sequence similarity thresholds. We followed the same procedure as we did to generate subsets of the SwissProt training sets with different sequence similarity thresholds: we used BLAST to calculate the pairwise sequence identity of each query protein to the protein clusters that form our main database. We removed clusters if they were more than 40%, 50%, 60%, 70%, 80%, and 95% similar to at least one of the query proteins in the test set. Since this operation removed or altered the content of the entries, we recalculated the intergenic distances for the remaining clusters and again split regions where the intergenic distance exceeded our 300-bp threshold, as we did when we created the main synteny database (Fig. 1B and C).

### 2.3 Comparison to published function prediction methods

#### 2.3.1 Comparison to broadly used function prediction methods as baseline

In our SwissProt benchmarks, we compared SAFPred to two conventional methods of function prediction: (i) BLAST (v. 2.12.0) (Altschul *et al.* 1990), widely used in the literature for comparisons to function prediction tools, and (ii) an HMM-based approach, as a more sophisticated baseline.

To predict function using the BLAST baseline, we transferred GO terms from significant BLAST hits also taking short sequences into account (e-value <1e-3, -task blastp-short) of a query protein with a predicted score of the value of the maximum sequence identity. As an alternative, we also used the GO term frequency-based approach (Zhou *et al.* 2019), but we found the maximum sequence identity scoring method performed better in our experiments.

To predict function using the HMM-based approach, we ran the hmmscan command from the HMMER package (Eddy 2011) with the flags “-E 1e-3—cpu 2—domtblout” against the Pfam database and applied the frequency-based approach to score transferred annotations, i.e. we transferred GO terms from all significant HMM hits (e-value <1e-3) to the query protein, using the frequency of a GO term (number of times it was observed among the significant hits) as the predicted score. To compare Pfam outputs quantitatively with those from other methods, we used Pfam2GO mapping tables (version date 5 December 2020) provided by the GO consortium to obtain GO terms corresponding to each Pfam ID in addition to the Pfam database (release 32.0) (Mitchell *et al.* 2015, Mistry *et al.* 2021). Because the Pfam database is independent of the training sets we created based on the SwissProt database, we could not evaluate its dependence on the similarity threshold examined for other tools.

#### 2.3.2 Comparison to a recent state-of-the-art deep learning method

We chose DeepGOPlus (v 1.0.1) (Kulmanov and Hoehndorf 2019) as a recent deep learning-based comparator in our experiments. A state-of-the-art tool, it uses a supervised approach where a deep convolutional neural network model is combined with a sequence homology-based method. We used the implementation provided by the authors and trained the model on the training sets in our experiments with the optimal values reported for the hyperparameters (Kulmanov and Hoehndorf 2019). We used the same training set for both the BLAST queries and the DeepGOPlus.

### 2.4 SAFPred algorithm

SAFPred combines two nearest neighbor (nn) methods: SAFPred-nn, which is based only on amino-acid level embeddings constructed from the SwissProt database, and SAFPred-synteny, which leverages syntenic relationships drawn from our bacterial synteny database.

In SAFPred-nn, we used the ESM-1b protein language model (which we will call ESM) (Rives *et al.* 2021) to represent SwissProt entries. To extract amino-acid level embedding vectors, we used bio_embeddings (v 0.2.2) (Dallago *et al.* 2021) with default settings. We obtained protein-level embeddings (1280 dimensional vectors for ESM) by averaging over individual amino acid embeddings. In preliminary work, we also used the ProtT5-XL-U50 model (Elnaggar *et al.* 2020), but found that embeddings from ESM performed better (Supplementary Material).

For each query protein, we identified nearest neighbors in the training set based on embedding vector similarity over a threshold, which we calculate separately for each query as the 99th percentile among all pairwise similarity values. We transferred GO terms from nearest neighbors with a score equal to their cosine similarity to the query protein. As the final prediction, we keep only the maximum score for each GO term transferred from the nearest neighbors. We use cosine similarity to determine the similarity between any two embedding vectors $\vec{e_{1}}$ and $\vec{e_{2}}$ defined as: $\mathrm{sim}(\vec{e_{1}},\vec{e_{2}})=(\vec{e_{1}}\cdot \vec{e_{2}})/(||\vec{e_{1}}||\cdot ||\vec{e_{2}}||)$, where $\vec{e_{1}}$ and $\vec{e_{2}}$ are both real-valued vectors, $\vec{e_{1}}\cdot \vec{e_{2}}$ represents the dot product between $\vec{e_{1}}$ and $\vec{e_{2}}$, and $\left||\vec{e_{i}}|\right|$ is the Euclidean norm of vector $\vec{e_{i}}$, for *i *=* *1, 2.

**Figure 2.**
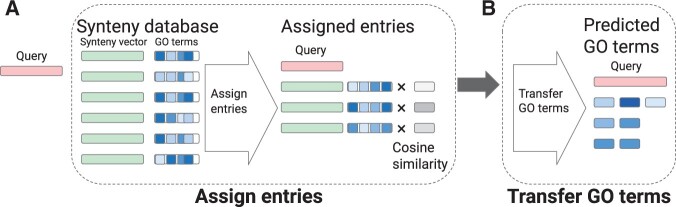
Overview of SAFPred-synteny algorithm: predicting GO terms of a query protein. (A) SAFPred-synteny assigns an entry (or multiple entries) to the query protein (red filled rectangle on the left) represented using embeddings from ESM-1b LM, based on cosine similarity. Consistent with Fig. 1, green rectangles show synteny embeddings paired with the corresponding GO term frequencies (blue rectangles). In this example, three entries that passed the threshold are assigned to the query, and their GO term frequencies are weighted by multiplying by the cosine similarity. (B) All GO terms from the assigned entries are transferred to the query, where the final predicted score of a GO term is the maximum of all the multiplied values for the term.

The SAFPred-synteny component comprises two main steps (Fig. 2): (i) assigning syntenic regions to a query from the precomputed synteny database, SAFPredDB (Fig. 2A) and (ii) transferring GO terms from SAFPredDB entries to the query (Fig. 2B). SAFPred-synteny follows the same nearest neighbor approach as SAPFred-nn to find the most suitable syntenic regions in SAFPredDB for each query point. In short, we calculate the pairwise cosine similarity between the query point and the average embedding vectors representing database entries. We assign a region to the query if the pairwise similarity between the region and query embeddings is greater than the 99th percentile among all pairwise similarity values.

In our current implementation, we do not have any restrictions on entries assigned to a query protein: given that the most suitable syntenic regions are picked among the same set of regions used to calculate the threshold, at least one region is assigned to each query point.

For all such entries assigned to the query, we also retrieve GO term frequencies. We transfer all GO terms found in the assigned entries using the frequency of the terms multiplied by the cosine similarity of the query point to the entry as the predicted score. For each GO term, the predicted score is the maximum of these values. As the final step in our algorithm, we normalize the predicted scores separately within three GO classes.

SAFPred combines the predictions from SAFPred-nn and SAFPred-synteny by taking the average of predicted scores. We also evaluated its two component predictors individually. Comparing all three methods side by side allowed us to assess the individual contributions from embeddings and our synteny database on SAFPred’s performance.

### 2.5 SwissProt benchmark evaluation

Using our SwissProt benchmarking datasets, we evaluated six protein prediction methods: two baselines (BLAST and Pfam), DeepGOPlus, SAFPred, and separately its two components SAFPred-nn and SAFPred-synteny, representing contributions from the embeddings representation and synteny, respectively. In order to make the outputs of all tools comparable to those of DeepGOPlus, we propagated the predicted GO term scores based on the GO hierarchy, as done previously (Kulmanov and Hoehndorf 2019). For each GO term, we assigned the highest predicted score from among its children. This additional postprocessing step was only implemented in our benchmarking comparisons across tools, and not in our function prediction across the *Enterococcus* genus.

We evaluated these function prediction methods as done for the CAFA challenges, using the maximum F1-score (*F*_max)_ and the minimum semantic distance (*S*_min)_ as described in (Zhou *et al.* 2019). We also report the coverage, defined as the percentage of test proteins annotated with at least one GO term at the threshold which maximizes the F1-score. We use leaf nodes in the GO hierarchy only, and remove all ancestor nodes between the leaves and the top of the tree.

### 2.6 Applying SAFPred to a diverse set of enterococcal genomes, including detailed analysis of pore-forming toxins

To demonstrate a practical application of SAFPred, we applied it to the *Enterococcus* diversity dataset. We ran SAFPred in default mode, comparing its output to that from three annotation approaches: (i) prokka (v. 1.14.6) (Seemann 2014), which runs multiple sequence homology-based function prediction tools; (ii) the Pfam database (release 32.0) (Paysan-Lafosse *et al.* 2023) using the hmmscan command from HMMER (v 3.3.2) (Eddy 2011); and (iii) eggNOG mapper (v 2.1.10) (Huerta-Cepas *et al.* 2018). All tools were run using default parameters; for HMMER and eggNOG, a significant hit was defined as having e-value <1e-3.

When examining potential novel *Enterococcus* pore-forming toxins, we performed additional analyses to assess the potential function of query proteins without experimental annotations: (i) we performed a large-scale structure search using the query protein against AlphaFoldDB and the Protein Data Bank (PDB); (ii) we examined their similarity to known pore-forming toxins found in *Enterococcus* or closely related genera (Supplementary Table S4), both in terms of structural similarity (using Foldseek), as well as in genomic context; and (iii) we assessed the presence of key structural elements, including N-terminal signal sequences, a common feature in most toxin sequences which guides toxin secretion and transportation outside the cell.

In order to compare syntenic relationships between predicted and known toxin genes, we examined five genes upstream and downstream of toxin genes predicted by SAFPred, as well as for the known delta toxin genes from Supplementary Table S4, *epx1* and *epx4* (Xiong *et al.* 2022).

To predict the structure of potential novel toxin genes identified by SAFPred, we used the Fold Sequence public server on ESMFold Atlas (Lin *et al.* 2023) which only allows input sequences shorter than 400 amino acids. For longer proteins, we used AlphaFold (Jumper *et al.* 2021) in monomer mode with default settings, using the Docker implementation. We used Foldseek (van Kempen *et al.* 2023) for both protein structure search against databases and structural alignment. While the structure database search was performed with default settings, we utilized both the global (—alignment-type 1) and local alignment options (—alignment-type 2) of Foldseek. Following the guidelines available for running Foldseek, we labeled alignments depending on their structural alignment score: highly significant (> 0.7), significant (0.6–0.7), nonrandom (0.5–0.6), or random (≤ 0.5). To account for large differences in the query and target sequence length, we required the alignment probability to be >0.8. We predicted the N-terminal signal sequences using the SMART server (Schultz *et al.* 1998).

## 3 Results

To improve gene function annotation for bacteria, we developed SAFPred, which combines state-of-the-art protein embeddings based on NLP algorithms with bacteria-specific information about gene function inferred from bacterial synteny collected in our database, SAFPredDB, which provides meaningful insight into gene function. This combination outperformed conventional gene function prediction tools and a recent state-of-the-art method, DeepGOPlus, on bacterial genes. We also demonstrated SAFPred’s performance on a real-world application where it identified potential novel variants of delta toxin in *Enterococcus*.

### 3.1 SAFPredDB: a database to leverage functional information from syntenic relationships across bacteria

To incorporate information about synteny into SAFPred, we constructed a large-scale database, SAFPredDB, of over 400 000 syntenic regions predicted from >45 000 representative genomes from across the bacterial kingdom (Methods). We validated SAFPredDB by comparison to the experimentally determined operons found in the conserved ODB (Okuda and Yoshizawa 2010), a similar online database. SAFPredDB is larger and more up-to-date than ODB, which is based on a smaller, curated list of experimentally determined operons from the literature. Overall, SAFPredDB is quantitatively similar to the conserved ODB, in terms of region length, number of genes in a region and intergenic distance within regions (Supplementary Figs S1–S3). SAFPredDB provides an extensive catalog of conserved patterns of synteny within the bacterial kingdom (Supplementary Table S1).

### 3.2 SAFPred outperforms other tools in function prediction for multiple bacterial species

To assess the performance of SAFPred in assigning GO terms to proteins, we first performed benchmarking on the SwissProt database, where only the proteins with at least one experimentally determined GO annotation were retained. We then created benchmarking datasets for five different bacterial species, dividing SwissProt entries into training and test sets, thus simulating the real-world scenario of annotating predicted proteins that lack exact matches to database entries.

We benchmarked SAFPred against three previously published tools, including (i) a baseline BLAST method; (ii) a basic HMM-based approach (HMMER); and (iii) a state-of-the-art deep learning method (DeepGOPlus) (Methods). We also compared SAFPred against its two component algorithms run separately, SAFPred-nn (which relies solely on protein embeddings) and SAFPred-synteny (which relies solely on a database of syntenic relationships from operons), allowing us to assess contributions of the two components. We performed benchmarking separately for three categories of GO terms, including Biological Process (BPO), Molecular Function (MFO), and Cellular Component (CCO), as these are known to present different challenges for annotation (Radivojac *et al.* 2013). Overall, SAFPred achieved the highest *F*_max_ scores across all five species, for all three GO categories, and on the full SwissProt benchmarking set, with *S. typhimurium* being the only exception. On this species, DeepGOPlus performed the best for BPO and MFO (Table 2 and Supplementary Table S7). We observed similar trends in prediction performance using *S*_min_ and the area under the precision/recall curve (Supplementary Tables S8 and S9). The SAFPRed-nn predictor used alone surpassed conventional tools, showing that protein embeddings, even in a simple unsupervised model, provided a better representation of protein sequence for GO term transfer than both the amino-acid sequence itself (BLAST baseline) and the HMM profiles (Pfam baseline) (Table 2 and Supplementary Table S7). This agreed with recent studies on eukaryotes (Heinzinger *et al.* 2022). SAFPred-synteny used alone performed substantially better than SAFPred-nn, highlighting the usefulness of incorporating syntenic information. SAFPred-synteny performed almost as well as the full SAFPred tool.

**Table 2.** *F*_max_ scores from our benchmarking for six different function prediction tools in the BPO category (MFO and CCO are shown in Supplementary Table S7), for each of five bacterial species in our full SwissProt benchmarking set.^a^

	**Bacterial species** ^b^				
	*EC*	*MT*	*BS*	*PA*	*ST*
Method	*F*_max_ scores for BPO				
BLAST	0.570	0.543	0.639	0.683	0.852
Pfam	0.610	0.513	0.582	0.579	0.579
DeepGOPlus	0.648	0.669	0.857	0.824	**0.928**
SAFPred-nn	0.646	0.636	0.828	0.797	0.880
SAFPred-synteny	0.872	0.837	**0.915**	0.928	0.903
SAFPred	**0.876**	**0.838**	**0.915**	**0.929**	0.902

a The highest *F*_max_ score in each column is shown in bold.

b EC, *Escherichia coli*; MT, *Mycobacterium tuberculosis*; BS, *Bacillus subtilis*; PA, *Pseudomonas aeruginosa*; ST, *Salmonella typhimurium.*

### 3.3 SAFPred surpasses existing tools for annotating distant homologs

We were particularly motivated to develop SAFPred to increase the number of annotations for the growing number of unannotated bacterial proteins, with few or no homologs in existing databases. To emulate gene function prediction of distant homologs, we designed additional benchmarking sets where the pairs of training and test sets were generated by stratifying the full SwissProt dataset based on the maximum sequence similarity allowed between protein sequences in the training and the test set.

**Figure 3.**
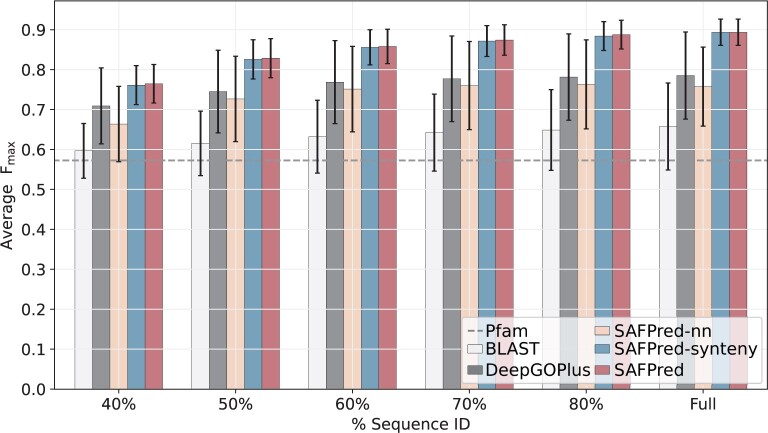
SAFPred outperformed conventional approaches to function prediction. Data is averaged across five bacterial species, for variable sequence identity to proteins in the training set (*x*-axis). Error bars show SDs. As the Pfam database is not dependent on the % sequence identity to the training set, a single value for *F*_max_ for the Pfam baseline is shown (dashed line).

As we did not observe any significant differences between the species examined, we report the average *F*_max_ values and SD for all five bacteria combined (BPO in Fig. 3). SAFPred was consistently the top-performing method. The difference in prediction performance (as measured by *F*_max)_ between SAFPred and all other methods was greater as the sequence similarity between the test and the training sequences (as well as the clusters in the synteny database) increased (Fig. 3).

Similar to the full datasets, we observed that protein embeddings (SAFPred-nn) far outperformed both conventional predictors, BLAST and Pfam, across the range of sequence similarities. Furthermore, as with the full datasets, we observed that SAFPred-synteny performed substantially better than SAFPred-nn, and almost as well as SAFPred, demonstrating the large contribution gained by adding information from synteny.

In addition, this benchmarking revealed that BLAST performance was surprisingly consistent across levels of shared homology, while the embeddings-based methods showed improvement in performance as similarity between the training and test sets increased. This trend held for not only the average *F*_max_ in the remaining two ontologies (MFO and CCO), but also for each bacterial species individually (Supplementary Tables S10–S14).

### 3.4 SAFPred provides more reliable predictions compared to other methods

Among the tools we benchmarked, the BLAST and HMM-based Pfam baselines had the lowest annotation coverages (i.e. the number of test genes that have at least one predicted GO term) on both the full SwissProt dataset and the sets with lower sequence similarity (Supplementary Tables S15 and S16). SAFPred emerged as the all-around top-performing method in terms of balancing precision and recall. Furthermore, we found that its prediction coverage was in line with other embeddings-based nn models on the full SwissProt benchmarking, although it occasionally lagged behind the state-of-the-art in terms of coverage on our other benchmark sets. Given that SAFPred achieved the best *F*_max_ values across the board, the drop in coverage means SAFPred’s predictions are more reliable compared to other methods.

We did observe that SAFPred’s coverage decreased slightly for test sets with lower similarity to the training set (Supplementary Table S16). In these benchmark tests, SAFPredDB is sparsely labeled due to a conservative annotation methodology (Supplementary Text), limiting the annotations that can be transferred based on synteny.

### 3.5 SAFPred identifies five potential novel pore-forming toxins among a diverse set of enterococcal genomes

A key goal in the development of SAFPred was predicting functions of unannotated genes in bacteria, including those associated with key bacterial features of clinical interest such as antimicrobial resistance and virulence. *Enterococcus* is a diverse genus of bacteria thought to inhabit the gastrointestinal tracts of all land animals. These organisms have an incredibly diverse functional repertoire, yet many of their predicted proteins are of unknown function (Lebreton *et al.* 2017, Schwartzman *et al.* 2023). Uncovering this rich functional diversity is of primary interest given the ubiquity and importance of this genus. Recent targeted searches have reported the discovery of several classes of novel toxins within diverse enterococcal species, including the discovery of a new family of pore-forming delta toxins in *E. faecalis*, *E. faecium*, and *E. hirae* (Xiong *et al.* 2022) and new botulinum toxins in *E. faecium* (Zhang *et al.* 2018). All of these newly discovered toxins exhibit low sequence similarity to known toxin sequences in other bacterial species.

Although the previous studies focused only on three clinically relevant species of *Enterococcus*, we hypothesized that similar toxins could also be found in other diverse, less well-studied species of *Enterococcus*, providing insights into other ecologies in which these toxins may be advantageous. Thus, to search for additional novel toxin genes across the *Enterococcus* genus, we applied SAFPred to a collection of 19 *Enterococcus* genomes, each representing a different species (Lebreton *et al.* 2017), including 16 species not examined by Xiong *et al.* or Zhang *et al.* We looked specifically for genes that were labeled with a GO term describing toxin activity and associated with the conserved genomic context of delta toxins (Xiong *et al.* 2022). SAFPred associated 59 genes with the single delta toxin operon from SAPdb, consisting of an enterotoxin and a putative lipoprotein cluster, found in the unrelated *Clostridium* and *Roseburia* species (Supplementary Table S5). Of these 59 genes, six were predicted by Pfam to be pore-forming toxins (e-value <1e-3 to PF01117 or PF03318), and three were annotated by Prokka as “lipoproteins” (Methods). The remaining 50 had no functional prediction prior to running SAFPred.

To explore their candidacy as delta toxin encoding, we evaluated each gene’s predicted protein structure and genomic context. Eleven (of 59) had structural similarity to known toxin structural folds (Foldseek alignment probability >0.8 and alignment score >0.5 to proteins in the AlphaFold and the Protein Data Bank (PDB) structure databases), including several with highly significant alignments (Supplementary Table S4 and Fig. S37). Of these eleven, five were not previously identified as having a toxin annotation by either Prokka or Pfam—these were detected only by SAFPred. All 11 contained signal peptides at similar positions as those in known bacterial toxins. The remaining 48 proteins without structural similarity had lower SAFPredDB rankings than the 11 with structural similarity (Supplementary Text).

In the absence of experimental annotations, we continued the analysis with the 11 candidate toxins identified by SAFPred to the known pore-forming delta toxin genes previously reported in *Enterococcus*, *epx1* and *epx4*; we compared their genomic context and their neighborhoods (Xiong *et al.* 2022). Seven of the 11 candidate toxin genes were most similar to *epx1* structures from *E. faecalis* and *S. aureus*, including five from *E. haemoperoxidus* BAA-382, and two from *E. pernyi* ATCC882. All had surrounding genes with some degree of structural similarity to genes within the known *epx1* genomic neighborhood, including two with highly significant matches (Supplementary Fig. S37A). Among the five putative toxin genes, the highest amino acid sequence identity to *epx1* was less than 40% (Supplementary Table S17). Furthermore, the gene neighborhood was conserved between the five candidates from *E. haemoperoxidus* BAA-382 (Supplementary Fig. S37).

Four of the 11 candidate toxin genes were most similar to the *E. hirae epx4* structure, including one gene from *E. haemoperoxidus* and three genes from *E. moraviensis* BAA-383 (Supplementary Fig. S37B). Among the four putative *epx4* genes, the maximum amino acid sequence similarity we observed to *epx4* was 60% (Supplementary Table S17). Similar to the *epx1* context, we observed that the neighboring genes of the new *epx4*-like toxins predicted by SAFPred were structurally similar to one another. Although some of the neighboring genes had lower Foldseek similarity scores, the neighborhoods had nonrandom similarity among themselves (scores ranging from 0.4 to 0.9).

## 4 Discussion

In this work, we introduce SAFPred, a novel synteny-aware, NLP-based function prediction tool for bacteria. SAFPred is distinguished from existing tools for annotating bacteria in two ways: (i) it represents proteins using embedding vectors extracted from state-of-the-art protein language models, and (ii) it incorporates additional functional information inferred from a protein’s genomic neighborhood, by leveraging conserved synteny across the entire bacterial kingdom, tabulated in our synteny database SAFPredDB. This allows SAFPred to identify coregulated genes that may be part of same functional pathways, but which have completely different sequence or protein structure. To our knowledge, SAFPred is the only bacterial gene function prediction tool with these two features.

While there have been successful uses of protein language models for gene function prediction in eukaryotes, these methods have not yet been extensively applied to bacteria. We confirmed that protein embeddings in SAFPred surpass conventional sequence homology-based tools, providing a better representation of genes to infer gene function (Table 2).

To assess SAFPred’s performance on bacteria, we designed a systematic, rigorous benchmarking framework based on the SwissProt database, where we further limited our training set according to its sequence similarity to the test set, in order to evaluate function predictors in the situation where there only distant homologs are known. We examined thresholds down to 40% sequence similarity, as previous work showed that proteins with identity >40% are likely to share functional similarity Pearson (2013). However, we know that BLASTp is an imperfect method for identifying homologous relationships for distantly related proteins, and we thus expect that distant homologues, including similarities in protein folds, will be present in our training set. A strength of our tool is its ability to identify functional relationships in distant homologs that sequence comparisons are unable to identify. As we have observed in our function prediction of enterococcal toxins, SAFPred can identify functional linkages to proteins with structural folds that share less than 30% sequence similarity. Thus, we expect that SAFPred’s performance would surpass conventional methods when sequence similarity is even lower than the thresholds we implemented in our SwissProt benchmarks. Future work will include benchmarks where we can evaluate SAFPred’s performance on unseen genes and assess its generalizability.

Although bacterial gene neighborhoods have been used previously for function prediction, this practice has mostly been manual and is absent from current automated annotation tools. We consistently achieved the best performance when synteny was used in conjunction with the embeddings representation within the SAFPred framework. Either component alone resulted in lower performance, while the biggest gain in prediction performance came from the use of synteny relationships. We demonstrate that conserved synteny and protein embeddings provide complementary information for predicting gene function, in particular when there are fewer homologs available (Fig. 3). We presume the overall improvement in prediction accuracy stems from both more accurate function prediction and homolog detection since SAFPred consistently outperforms other methods, even when the sequence similarity between training and test set is low. In future work, a different experiment should be designed to study homolog detection specifically in addition to expanding the set of comparator tools to provide more insight.

We demonstrated that SAFPred improves homolog detection for 19 diverse enterococcal species. Following the recent discovery of several types of novel toxin genes in enterococci, we focused on toxin discovery. SAFPred predicted 11 candidate delta toxin genes, which showed low sequence similarity to known toxins (<30%) but significant structural homology to known toxin protein structural folds. Several of these candidates also shared similar genomic neighborhood patterns with those of known toxin genes. Although six of these candidate toxins could also be identified based on their Pfam domains, five of these could not be annotated using any of the existing gene prediction tools. These five genes are strong candidates for further experimental validation of their toxin activities. SAFPred also identified 48 additional genes with functional linkages to toxin operons, but without structural homology to known toxins. The function of these genes should be investigated in future studies as well.

One limitation of SAFPred is its reliance on a predicted synteny database, which may contain syntenic linkages that do not share a function, in addition to actual operons. Also, in the absence of ground truth, both the operon predictions and the functions we assigned to these operons are limited by the existing databases (Supplementary Text). To minimize false positives, we adopted a conservative approach which in turn resulted in a sparsely annotated training set, lowering the prediction coverage of SAFPred (Supplementary Tables S15 and S16). One way to alleviate this problem is to routinely pick unlabeled entries from our database, prioritizing the most common ones, to perform experiments and identify their functions. With each new experimental annotation available, additional entries can be labeled. We expect this iterative approach to rapidly increase the number of labeled entries in the database.

Another limitation of the current version of SAFPredDB is its focus on broadly conserved patterns; it represents synteny across the entire bacterial kingdom. Since our goal was to develop an all-purpose bacterial gene annotation tool, we deliberately designed our database to be inclusive and to cover as many syntenic regions as possible. Thus, syntenic patterns or operons associated with rare traits, or functional pathways unique to novel species are not present in the default SAFPredDB, but are straightforward to add for specific analyses, as SAFPredDB can be tailored and reconstructed using the latest releases of its source databases. We provide scripts to customize and keep it up to date. For instance, a version of SAFPredDB incorporating metagenomic data could be used to study new functions in uncultured bacteria. Or, to design an all-purpose annotation pipeline for prokaryotes, SAFPredDB could be expanded to cover the diversity of prokaryotes. Although we used only GO terms to describe gene function, the new database could incorporate additional features, such as enzymatic activity and pathways to better capture functional traits. Finally, different representations of synteny vectors in the database, other than taking the average of embeddings, could be explored.

Currently, SAFPred assigns every query gene the same number of entries, equal to 1% of all entries available in the dataset, in order to be as inclusive as possible in learning about unannotated genes. To help disambiguate real matches from false positives, SAFPred reports a rank for each of the matching entries based on their similarity to the query. Although we have not determined whether a universal ranking threshold exists, our detailed examination of toxin operons in *Enterococcus* suggested this ranking can be a reliable proxy for confidence. While SAFPred reported 48 additional genes associated with the delta toxin operon, the delta toxin operon ranked among the top two entries for only the 11 candidate genes that showed structural similarity to the toxin fold. Thus, the order of assigned entries could be used as a proxy to infer confidence.

We demonstrated that conserved synteny and protein embeddings both provide useful information for predicting the protein function. SAFPred outperforms conventional sequence-based bacterial genome annotation pipelines, as well as more sophisticated HMM-based approaches and more recently developed deep learning methods. SAFPred can not only infer beyond the linear sequence, at the level of protein fold, but it can also successfully utilize conserved synteny among bacterial species to predict gene function.

## Supplementary Material

btae328_Supplementary_Data

## Data Availability

All data used for the analyses in this article are publicly available in the Uniprot database and the Assembly Database at NCBI (accession IDs of *Enterococcus* assemblies listed in Supplementary Table S5). The code and the scripts developed in this work are public at https://github.com/AbeelLab/safpred.

## References

[btae328-B1] Altschul SF , Gish W, Miller W et al Basic local alignment search tool. J Mol Biol 1990;215:403–10. 2231712 10.1016/S0022-2836(05)80360-2

[btae328-B2] Ashburner M , Ball CA, Blake JA et al Gene ontology: tool for the unification of biology. Nat Genet 2000;25:25–9. 10802651 10.1038/75556 PMC3037419

[btae328-B3] Dallago C , Schütze K, Heinzinger M et al Learned embeddings from deep learning to visualize and predict protein sets. Curr Protoc 2021;1:e113. 33961736 10.1002/cpz1.113

[btae328-B4] de Daruvar A , Collado-Vides J, Valencia A et al Analysis of the cellular functions of escherichia coli operons and their conservation in bacillus subtilis. J Mol Evol 2002;55:211–21. 12107597 10.1007/s00239-002-2317-1

[btae328-B5] Eddy SR. Accelerated profile hmm searches. PLoS Comput Biol 2011;7:e1002195. 22039361 10.1371/journal.pcbi.1002195 PMC3197634

[btae328-B6] Elnaggar A , Heinzinger M, Dallago C et al ProtTrans: Toward understanding the language of life through self-supervised learning. *IEEE Trans Pattern Anal Mach Intell* 2022;44:7112–27. 10.1109/TPAMI.2021.3095381. 34232869

[btae328-B7] Heinzinger M , Elnaggar A, Wang Y et al Modeling aspects of the language of life through transfer-learning protein sequences. BMC Bioinformatics 2019;20:723. 31847804 10.1186/s12859-019-3220-8 PMC6918593

[btae328-B8] Heinzinger M , Littmann M, Sillitoe I et al Contrastive learning on protein embeddings enlightens midnight zone. NAR Genom Bioinform 2022;4:lqac043. 35702380 10.1093/nargab/lqac043 PMC9188115

[btae328-B9] Hoarfrost A , Aptekmann A, Farfañuk G et al Deep learning of a bacterial and archaeal universal language of life enables transfer learning and illuminates microbial dark matter. Nat Commun 2022;13:2606. 35545619 10.1038/s41467-022-30070-8 PMC9095714

[btae328-B10] Huerta-Cepas J , Szklarczyk D, Heller D et al eggNOG 5.0: a hierarchical, functionally and phylogenetically annotated orthology resource based on 5090 organisms and 2502 viruses. Nucleic Acids Res 2018;47:D309–14. 10.1093/nar/gky1085 PMC6324079 30418610

[btae328-B11] Jumper J , Evans R, Pritzel A et al Highly accurate protein structure prediction with AlphaFold. Nature 2021;596:583–9. 34265844 10.1038/s41586-021-03819-2 PMC8371605

[btae328-B12] Kulmanov M , Hoehndorf R. DeepGOPlus: improved protein function prediction from sequence. Bioinformatics 2019;36:422–9. 10.1093/bioinformatics/btz595 PMC9883727 31350877

[btae328-B13] Lebreton F , Manson AL, Saavedra JT et al Tracing the enterococci from paleozoic origins to the hospital. Cell 2017;169:849–61.e13. 28502769 10.1016/j.cell.2017.04.027 PMC5499534

[btae328-B14] Li W , Godzik A. CD-hit: a fast program for clustering and comparing large sets of protein or nucleotide sequences. Bioinformatics 2006;22:1658–9. 16731699 10.1093/bioinformatics/btl158

[btae328-B15] Li X , Chen H, Li J et al Gene function prediction with gene interaction networks: a context graph kernel approach. IEEE Trans Inf Technol Biomed 2009;14:119–28. 19789115 10.1109/TITB.2009.2033116

[btae328-B16] Lin Z , Akin H, Rao R et al Evolutionary-scale prediction of atomic-level protein structure with a language model. Science 2023;379:1123–30. 36927031 10.1126/science.ade2574

[btae328-B17] Littmann M , Heinzinger M, Dallago C et al Embeddings from deep learning transfer go annotations beyond homology. Sci Rep 2021;11:1160–14. 33441905 10.1038/s41598-020-80786-0 PMC7806674

[btae328-B18] Mahlich Y , Steinegger M, Rost B et al HFSP: high speed homology-driven function annotation of proteins. Bioinformatics 2018;34:i304–12. 29950013 10.1093/bioinformatics/bty262 PMC6022561

[btae328-B19] Mahlich Y , Zhu C, Chung H et al Learning from the unknown: exploring the range of bacterial functionality. Nucleic Acids Res 2023;51:10162–75. 37739408 10.1093/nar/gkad757 PMC10602916

[btae328-B20] Makrodimitris S , Reinders M, van Ham R et al A thorough analysis of the contribution of experimental, derived and sequence-based predicted protein-protein interactions for functional annotation of proteins. PLoS One 2020;15:e0242723. 33237964 10.1371/journal.pone.0242723 PMC7688180

[btae328-B21] Mistry J , Chuguransky S, Williams L et al Pfam: the protein families database in 2021. Nucleic Acids Res 2021;49:D412–9. 33125078 10.1093/nar/gkaa913 PMC7779014

[btae328-B22] Mitchell A , Chang H-Y, Daugherty L et al The interpro protein families database: the classification resource after 15 years. Nucleic Acids Res 2015;43:D213–21. 25428371 10.1093/nar/gku1243 PMC4383996

[btae328-B23] Okuda S , Yoshizawa AC. ODB: a database for operon organizations, 2011 update. Nucleic Acids Res 2010;39:D552–5. 21051344 10.1093/nar/gkq1090 PMC3013687

[btae328-B24] Parks DH , Chuvochina M, Rinke C et al GTDB: an ongoing census of bacterial and archaeal diversity through a phylogenetically consistent, rank normalized and complete genome-based taxonomy. Nucleic Acids Res 2021;50:D785–94. 10.1093/nar/gkab776 PMC8728215 34520557

[btae328-B25] Paysan-Lafosse T , Blum M, Chuguransky S et al InterPro in 2022. Nucleic Acids Res 2023;51:D418–27. 36350672 10.1093/nar/gkac993 PMC9825450

[btae328-B26] Pearson WR. An introduction to sequence similarity (“homology”) searching. Curr Protoc Bioinformatics 2013;Chapter 3:3.1.1–1.8. 10.1002/0471250953.bi0301s42 PMC3820096 23749753

[btae328-B27] Radivojac P , Clark WT, Oron TR et al A large-scale evaluation of computational protein function prediction. Nat Methods 2013;10:221–7. 23353650 10.1038/nmeth.2340 PMC3584181

[btae328-B28] Rives A , Meier J, Sercu T et al Biological structure and function emerge from scaling unsupervised learning to 250 million protein sequences. Proc Natl Acad Sci USA 2021;118:e2016239118. 33876751 10.1073/pnas.2016239118 PMC8053943

[btae328-B29] Schultz J , Milpetz F, Bork P et al SMART, a simple modular architecture research tool: identification of signaling domains. Proc Natl Acad Sci USA 1998;95:5857–64. 9600884 10.1073/pnas.95.11.5857 PMC34487

[btae328-B30] Schwartzman JA , Lebreton F, Salamzade R et al Global diversity of enterococci and description of 18 previously unknown species. *Proc Natl Acad Sci U S A* 2024;121:e2310852121. 10.1073/pnas.2310852121. 38416678 PMC10927581

[btae328-B31] Seemann T. Prokka: rapid prokaryotic genome annotation. Bioinformatics 2014;30:2068–9. 24642063 10.1093/bioinformatics/btu153

[btae328-B32] The UniProt Consortium. UniProt: a worldwide hub of protein knowledge. Nucleic Acids Res 2018;47:D506–15. 10.1093/nar/gky1049 PMC6323992 30395287

[btae328-B33] Van Kempen M , Kim SS, Tumescheit C et al Fast and accurate protein structure search with foldseek. *Nat Biotechnol* 2024;42:243–6. 10.1038/s41587-023-01773-0. 37156916 PMC10869269

[btae328-B34] Xiong X , Tian S, Yang P et al Emerging enterococcus pore-forming toxins with MHC/HLA-I as receptors. Cell 2022;185:1157–71.e22. 35259335 10.1016/j.cell.2022.02.002 PMC8978092

[btae328-B35] Yao S , You R, Wang S et al NetGO 2.0: improving large-scale protein function prediction with massive sequence, text, domain, family and network information. Nucleic Acids Res 2021;49:W469–75. 34038555 10.1093/nar/gkab398 PMC8262706

[btae328-B36] You R , Zhang Z, Xiong Y et al GOLabeler: improving sequence-based large-scale protein function prediction by learning to rank. Bioinformatics 2018;34:2465–73. 29522145 10.1093/bioinformatics/bty130

[btae328-B37] Zhang S , Lebreton F, Mansfield MJ et al Identification of a botulinum neurotoxin-like toxin in a commensal strain of enterococcus faecium. Cell Host Microbe 2018;23:169–76.e6. 29396040 10.1016/j.chom.2017.12.018 PMC5926203

[btae328-B38] Zhou N , Jiang Y, Bergquist TR et al The CAFA challenge reports improved protein function prediction and new functional annotations for hundreds of genes through experimental screens. Genome Biol 2019;20:244. 31744546 10.1186/s13059-019-1835-8 PMC6864930

