# Supplementary material for: SAFPred: synteny-aware gene function prediction for bacteria using protein embeddings
Source: Bioinformatics. 2024 May 22;40(6):btae328. doi: 10.1093/bioinformatics/btae328 (PMC11147799; doi:10.1093/bioinformatics/btae328)
Supplement: btae328_Supplementary_Data [file btae328_supplementary_data.pdf]

# Supplementary Material for SAFPred: Synteny-aware gene function predictor for bacteria using protein embeddings

Aysun Urhan[1,2], Bianca-Maria Cosma[1], Ashlee M. Earl[2], Abigail L. Manson[2] and Thomas Abeel[1,2,*]

[1]Delft Bioinformatics Lab, Delft University of Technology Van Mourik, Broekmanweg 6, 2628 XE, Delft, The Netherlands and

[2]Infectious Disease and Microbiome Program, Broad Institute of MIT and Harvard, 415 Main Street, Cambridge, MA, 02142, US.

[8]To whom correspondence should be addressed.

# Table of Contents

**Table of Contents** 1
**Supplementary Text: SwissProt Dataset for Benchmarking** 2
**Supplementary Text: Detailed examination of SAFPredDB** 3
Comparison of SAFPredDB entries to experimentally determined operons 3
Annotating SAFPredDB entries 6
Analysis of false positive enterococcal toxin predictions 8
**Supplementary Tables** 10
**Supplementary Figures** 22
**References** 28

# Supplementary Text: SwissProt Dataset for Benchmarking

SAFPred was developed to improve gene function prediction in bacteria, and to evaluate our method on this specialized task we built a bacterial benchmarking dataset based on the SwissProt database (The UniProt Consortium, 2018).

SwissProt Database (release 2021-04, retrieval date 10 November 2021) forms the basis of our benchmarking dataset. First, we limited the entries to proteins of length [40,1000] to minimize the effect of protein length on our study. Then, we restricted the dataset to include only experimental GO annotations, we retained SwissProt entries with at least one GO term with the evidence codes EXP, IDA, IPI, IMP, IGI, IEP, HTP, HDA, HMP, HGI, HEP, IBA, IBD, IKR, IRD, IC, and TAS. Finally, we clustered the proteins using CD-HIT at 95% sequence similarity threshold to reduce redundancy (Li and Godzik, 2006). At the end of this procedure, we had 107,818 proteins in total. This dataset reflects the full scope of organisms present in SwissProt, with 2005 unique tax IDs, 737 of which belong to bacteria including proteobacteria, terrabacteria, archaea and spirochaetes.

To create our bacterial benchmark datasets, we selected the five bacterial organisms that had the largest number of entries in SwissProt: *Escherichia coli* (*EC*), *Mycobacterium tuberculosis* (*MT*), *Bacillus subtilis* (*BS*), *Pseudomonas aeruginosa* (*PA*), and *Salmonella typhimurium* (*ST*). For each bacteria, we obtained the “Full” dataset by reserving all proteins that belong to the organism to the test set. I.e., the “Full” EC dataset comprises a test set made up of only *E. coli* genes (3454 entries), and a training set of the remainder entries from SwissProt (104377 entries).

Next, to evaluate SAFPred on more challenging prediction scenarios where the goal is to predict gene function with few or no homologs in the existing databases, we created additional train/test set pairs for each bacteria. We removed proteins from the training set if they were more than 40%, 50%, 60%, 70% and 80% similar to any protein in the corresponding test set. This led to 6 train/test pairs for bacteria; the test set size remained constant, while the training set decreases in size as the sequence similarity threshold increases because more proteins are removed from the training set. In total, we had 30 train/test pairs (Table 1 in the manuscript).

In addition to the training set derived from SwissProt, SAFPred relies on the synteny database, SAFPredDB. Thus, we followed the same procedure to create additional databases corresponding to the preset sequence similarity threshold. We ran BLASTp on all genes in

SAFPredDB, and removed members from the database if they were more than 40%, 50%, 60%, 70%, 80% and 95% similar to any protein in the corresponding test set. This operation altered the contents of the entries, thus we recalculated the intergenic distance for each entry in SAFPredDB and split the regions if the intergenic distance exceeded our 300bp threshold.

# Supplementary Text: Detailed examination of SAFPredDB

## Comparison of SAFPredDB entries to experimentally determined operons

The goal of constructing our own large-scale synteny database (SAFPredDB) was to model the landscape of functions encapsulated within bacterial synteny in sufficient detail to be able to leverage this resource to improve bacterial function prediction. In this section, we validate SAFPredDB as an adequate database of conserved syntenic regions by comparing it to a smaller operon database published previously titled Operon Database (ODB v4) (Okuda and Yoshizawa 2010).

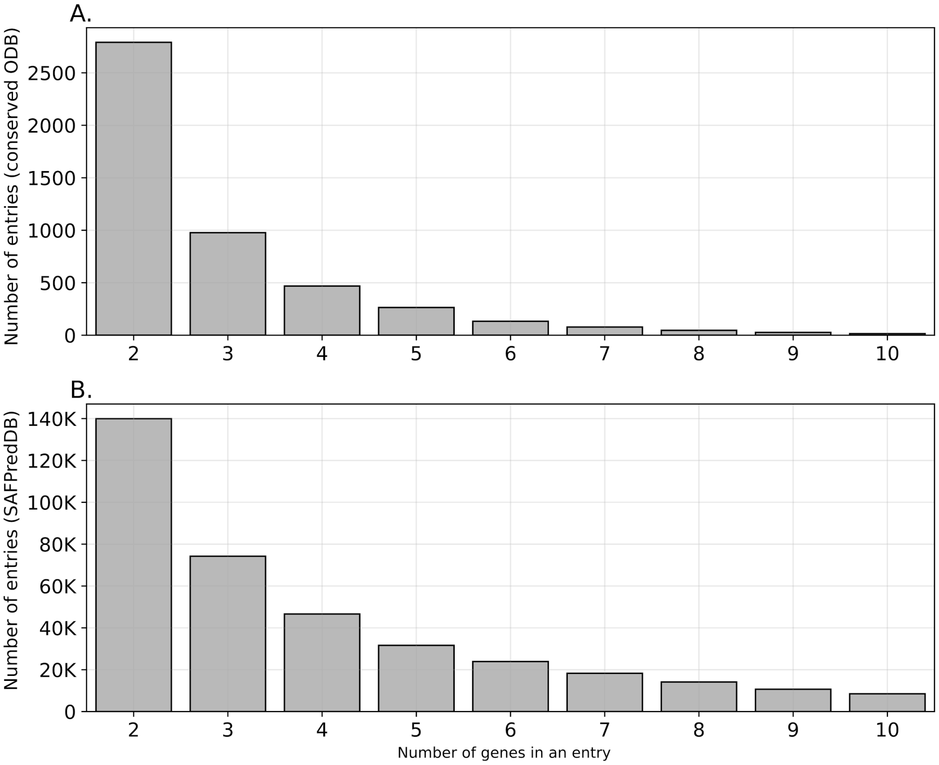


Fig S1. The entries in SAFPredDB have a similar size distribution to those in ODB: A. operons in ODB. B. entries in SAFPredDB. Only non-singleton entries are shown.

In constructing SAFPredDB, we used information from ODB to refine thresholds such as region length, number of genes in a region, and intergenic distance between adjacent genes in a region (Methods). ODB contains a curated list of experimentally determined operons obtained from the literature. The conserved operon database from ODB, referred to here as “ODB Conserved”, is essentially an expansion on their known, experimentally determined operons, where the additional predicted operon instances were identified in additional genomes. Overall, our synteny database was similar to the ODB Conserved database, in terms of region length, number of genes in an entry and intergenic distance within entries (Fig S1-S3). Thus, with SAFPredDB we provide a database even more extensive than ODB Conserved, representing a more up-to-date and broader range of diversity within the bacterial kingdom.

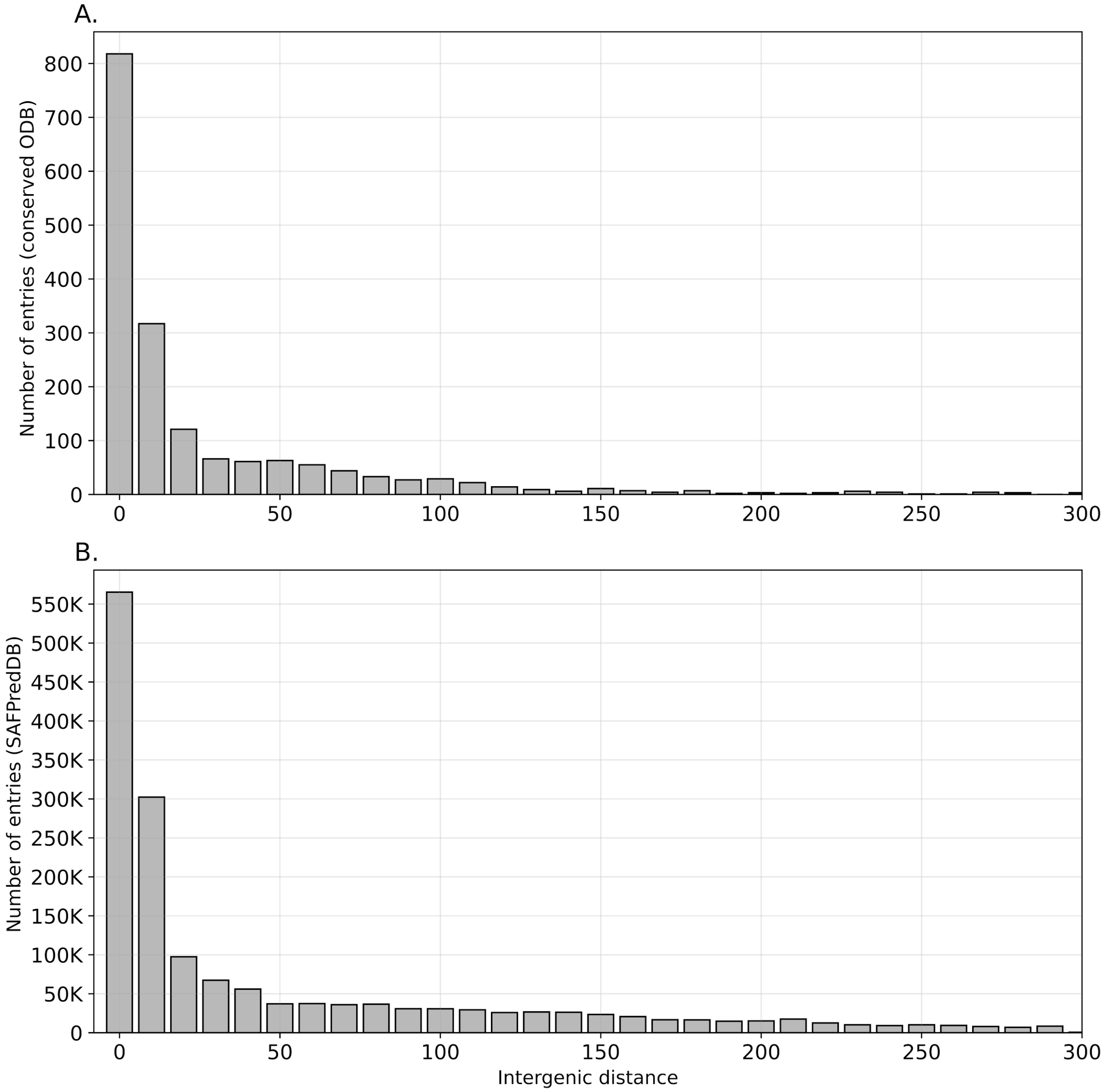

Fig S2. The entries in SAFPredDB have similar intergenic distance (bp) distribution to those in ODB: A. operons in ODB B. entries in SAFPredDB. Only non-singleton entries are shown.

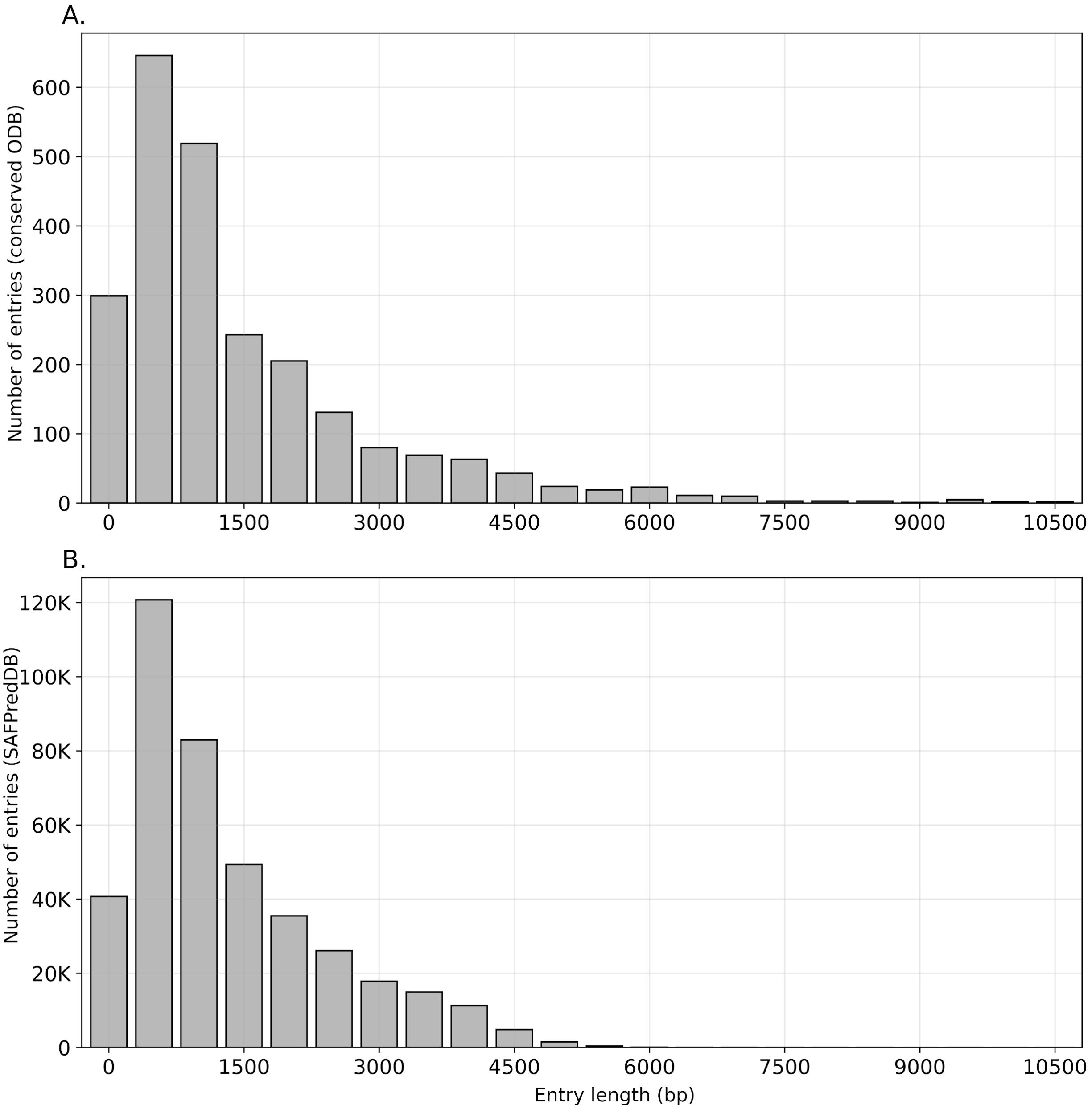


Fig S3. The entries in SAFPredDB have similar entry length distribution to those in ODB. A. operons in ODB. B. entries in SAFPredDB. Only non-singleton entries are shown.

With our approach, we also demonstrated that we could either partially or completely predict existing bacterial operons accurately. In order to assess whether we could reconstruct experimentally-determined operons in ODB for *E. coli*, based on our predicted regions in SAFPredDB, we extracted ODB operons containing at least one *E. coli* genes (2845 total operons, including 1071 non-singleton operons). We compared this to the predicted syntenic regions containing at least one *E. coli* gene in SAFPredDB, including 15,618 non-singleton entries, of

which 87% (13,610 entries) partially or fully overlap with the known ODB operons. Full concordance is not expected, as our database is substantially larger and encompasses far more genes. In addition, our database is inherently redundant; a single experimentally known operon from ODB can be split into multiple entries in SAFPredDB. Thus, it is highly likely that the remaining 2008 entries (23% of SAFPredDB) we predicted in addition to the ODB operons can be collapsed into a smaller number of actual syntenic regions or operons.

We selected a few predictions showcasing the accuracy of SAFPredDB in reproducing experimentally known operons (Table S1). Although SAFPredDB often accurately reproduces known operons, it can sometimes capture additional genes in the flanking regions (i.e.IS3 family transposase genes flanking the relBEF toxin-antitoxin system in Table S1). Although this can give us additional information about conserved mechanisms related to the operon's mobilization, this can also lead to false positive annotation transfers using SAFPredDB.

Table S1. Our synteny prediction algorithm can reproduce experimentally known operons in *E. coli*. Three example entries from SAFPredDB are shown, together with their corresponding operons in ODB, which were manually curated based on experimental studies.

| **Predicted operons in SAPdb** | | | | **Known operons in ODB** | | |
|---|---|---|---|---|---|---|
| **SAPdb Operon ID** | **Species** | ***E.coli gene*** | **Annotation** | **ODB Operon ID** | **Name** | **Definition** |
| 62671 | *Escherichia* , *Shigella*, *Citrobacter* | b4460 | L-arabinose transport system permease protein AraH | KO03244 | araFGH | High-affinity L-arabinose transport |
| | | b1900 | Arabinose import ATP-binding protein AraG (EC 7.5.2.12) | | | |
| | | b1901 | L-arabinose-binding periplasmic protein (ABP) | | | |
| 62674 | *Escherichia* , *Shigella*, *Bacillus* | b1879 | Flagellar biosynthetic protein FlhB | KO03228 | flhAB | Flagellar biosynthesis |
| | | b1880 | Flagellar biosynthesis protein FlhA | | | |
| 62755 | *Citrobacter*, *Enterobacter*, *Klebsiella*, *Leclercia*, *Escherichia*, | - | IS3 family transposase | - | - | |
| | | - | IS3 family transposase | - | - | |
| | | b1562 | Toxic protein HokD | KO03197 | relBEF | toxin-antitoxin system |
| | | b1563 | mRNA interferase toxin RelE (EC 3.1.-.-) (Endoribonuclease RelE) (Toxin RelE) | | | |
| | | b1564 | Antitoxin RelB | | | |

## Annotating SAFPredDB entries

Although GTDB is the largest catalog of representative bacterial genomes, it does not include experimental annotations for the gene sequences. Thus, we assigned function to the SAFPredDB entries by transferring GO terms from similar protein sequences in the SwissProt database,

using thresholds on both the pairwise identity and significance (Methods). When transferring GO terms to SAFPredDB entries, we aimed to balance minimizing false positives while retaining as many annotations as possible, in order to avoid having a database that was too sparsely annotated to be useful for predicting gene function. In our full SwissProt dataset experiments, 95.6% of the non-singleton entries (388,377 out of 406,293) were annotated with at least one GO term; however, in remote homology experiments, SAFPredDB was a lot more sparse in annotations. Since we removed training proteins that had more than a certain predefined level of sequence homology to any of the test proteins in order to create the remote homology datasets, there were only rare proteins with no homologs left in the database. Given that such rare proteins are less likely to be studied, annotated or even carry out any function within the cell, this loss of functional information was expected. Since SAFPredDB is a major source of annotation that SAFPred relies on, SAFPred's prediction coverage also suffers from this loss of information, and we observed that the difference in coverage between SAFPred and DeepGOPlus widens as more annotations are lost (Table S13 and S14).

**Table S2.** GO term annotations in SAFPredDB were more sparse in the remote homology detection experiments. Percentage of entries in SAFPredDB that were annotated with at least one GO term for each GO term category and every organism in our SwissProt benchmarks.

| | 40 | 50 | 60 | 70 | 80 | Full |
|---|---|---|---|---|---|---|
| **Organism** | **Biological process** | | | | | |
| *E. coli* | 74.66% | 80.23% | 82.33% | 83.84% | 84.16% | 84.83% |
| *M. tuberculosis* | 80.32% | 83.55% | 84.65% | 85.07% | 85.21% | 85.18% |
| *B. subtilis* | 79.30% | 83.82% | 84.63% | 85.17% | 85.09% | 85.15% |
| *P. aeruginosa* | 79.21% | 82.10% | 82.79% | 83.89% | 84.34% | 85.16% |
| *S. typhimurium* | 83.56% | 84.44% | 84.80% | 84.96% | 85.03% | 85.16% |
| | **Molecular function** | | | | | |
| *E. coli* | 77.37% | 83.08% | 85.77% | 87.24% | 87.84% | 88.38% |
| *M. tuberculosis* | 83.08% | 86.40% | 87.57% | 88.13% | 88.38% | 88.59% |
| *B. subtilis* | 81.55% | 85.73% | 87.51% | 88.06% | 88.48% | 88.58% |
| *P. aeruginosa* | 82.03% | 84.94% | 86.35% | 87.47% | 87.97% | 88.59% |
| *S. typhimurium* | 87.27% | 87.90% | 88.24% | 88.45% | 88.49% | 88.59% |
| | **Cellular component** | | | | | |
| *E. coli* | 69.11% | 75.94% | 79.57% | 81.25% | 81.94% | 82.79% |
| *M. tuberculosis* | 76.02% | 80.00% | 81.83% | 82.49% | 82.82% | 83.06% |
| *B. subtilis* | 75.50% | 79.87% | 81.98% | 82.76% | 82.97% | 83.06% |
| *P. aeruginosa* | 75.71% | 78.92% | 80.86% | 81.92% | 82.44% | 83.05% |

| | | | | | | |
|---|---|---|---|---|---|---|
| *S. typhimurium* | 81.37% | 82.26% | 82.61% | 82.84% | 82.91% | 83.06% |

Furthermore, we note that annotations were transferred at uneven rates for different categories of GO terms, consistent with previous findings in the literature. When sequence homology is used as the basis for transferring annotation, GO terms in the Molecular Function (MFO) category are more likely to be transferred than those for Biological Process (BPO) and Cellular Component (CCO), as MFO can more readily be modeled using the primary sequence, or features derived from it (Zhou et al. 2019). We also report that the number of entries annotated with at least one GO term is the largest for the MFO category, even though the total number of MFO terms available in the SwissProt dataset is significantly smaller than that of BPO (Table S3).

**Table S3.** Annotation statistics and information content (IC) of entries in SAFPredDB. GO terms in the Molecular Function (MFO) category are more likely to be transferred when using sequence homology for annotation transfer.

| **Annotation statistic** | **BPO**[a] | **MFO**[b] | **CCO**[c] |
|---|---|---|---|
| # of annotated entries (%) | 268,773 (70%) | 311,424 (80%) | 268,359 (70%) |
| Range of # of GO terms in an annotated entry | [1, 41] | [1, 17] | [1, 20] |
| Average # of GO terms per gene in an annotated entry | 0.556 | 0.543 | 0.456 |
| Average IC of GO terms in an annotated entry | 10.73 | 8.786 | 6.022 |
| Average IC of GO terms per gene in an annotated entry | 3.345 | 2.711 | 1.762 |
| Total # of GO terms in the SwissProt database | 16281 | 6308 | 2565 |

[a]Biological Process
[b]Molecular Function
[c]Cellular Component

## Analysis of false positive enterococcal toxin predictions

To detect novel toxins, we first collected known pore-forming toxin genes observed in Enterococcus or closely related genera. Table S4 lists all such toxins, along with their Uniprot IDs and PDB identifiers, if their structure is available in the PDB database.

**Table S4.** Known pore-forming toxin genes found in *Enterococcus* or closely related genera. Protein structures for all but two genes have been solved experimentally and were downloaded from PDB. For Leucotoxin and Alveolysin, AlphaFold was used to predict their structure since they were not found in the databases.

| **Gene** | **Sequence ID**[1] | **Structure ID**[2] | **Species** |
|---|---|---|---|
| Alpha-hemolysin | P09616 | 3m3r | *S. aureus* |
| Alveolysin | P23564 | - | *P. alvei* |
| Cytolysin | P19247 | 4owl | *V. vulnificus* |
| Gamma-hemolysin component A | P0A071 | 3b07 | *S. aureus* |
| Gamma-hemolysin component B | A0A0H3JX61 | 4p1x | *S. aureus* |

| | | | |
|---|---|---|---|
| Heat-labile enterotoxin B chain | P01558 | 3ziw | *C. perfringens* |
| Hemolysin | P09545 | 1xez | *V. cholerae* |
| Leucotoxin LukE | O54081 | - | *S. aureus* |
| Pore-forming toxin *epx1* | WP_104660001.1 | 7t4e | *E. faecalis* |
| Pore-forming toxin *epx4* | WP_053766529.1 | 7t4d | *E. hirae* |

[1]Uniprot IDs for the first 8 rows, and NCBI protein IDs for the last two rows.
[2]PDB identifiers.

Among the 10 toxin genes, we located the heat-liable enterotoxin B chain in an entry in SAFPredDB. Forty-eight genes were associated with the toxin operon in SAFPredDB, but did not have structural similarity to a known toxin protein fold. The SAFPredDB entry codes for two membrane-associated proteins: 1) the toxin gene itself, and 2) another membrane protein of unknown function. Based on FoldSeek search results, 33 of these 48 genes appeared to perform other functions related to the cell membrane, involving signaling, secretion, and pore formation. To gain further insight into why these 48 additional genes without toxin structural folds were matched to the delta toxin entry in SAFPredDB, we compared the Euclidean similarity of embeddings vectors extracted from all 59 genes to the embedding vector of the known delta toxin operon (Table S5). While we did not find any significant differences in terms of the absolute similarity values between those 11 that had structural similarity to the delta toxin operon and the rest, we noted that the SAFPredDB toxin entry was consistently ranked in the top two among all SAFPredDB entries assigned to the 11 likely toxins, whereas for the remaining 48 genes this was not the case. This suggests that the ranking could be used in future versions of SAFPred as a proxy to infer confidence in region assignments and to reduce false positives.

**Table S5.** SAFPred associated 59 enterococcal proteins with the delta toxin entry from SAFPredDB (entry ID 395786), found in *Clostridium* and *Roseburia*. The contents of this entry are shown here.

| Entry | Cluster | Gene | Uniprot ID |
|---|---|---|---|
| 395786 | 75445122 | Heat-labile enterotoxin B chain | P01558 |
| | 84731674 | Uncharacterized lipoprotein YsaB | Q83J37 |

# Supplementary Tables

**Supplementary Table. S6.** Genome metadata and assembly statistics of the 19 *Enterococcus* genomes used in the *Enterococcus* diversity dataset

| Species | Clade[1] | NCBI Accession | Genome size (bp) | N50 (bp) | Number of components[2] | Completeness[3] | Contamination[4] | Number of CDS[5] |
|---|---|---|---|---|---|---|---|---|
| *E. caccae* | I | GCA_000407145.1 | 3551922 | 1036503 | 7 | 98.99 | 1.01 | 3244 |
| *E. haemoperoxidus* | I | GCA_000407165.1 | 3581919 | 2345301 | 2 | 100 | 1.52 | 3207 |
| *E. moraviensis* | I | GCA_000407445.1 | 3586110 | 878836 | 6 | 98.99 | 0 | 3337 |
| *E. durans* | II | GCA_000407265.1 | 3170574 | 972116 | 11 | 98.99 | 0 | 3031 |
| *E. hirae* | II | | 2882665 | 676112 | 6 | 98.99 | 0 | 2667 |
| *E. pernyi* | II | GCA_000407465.1 | 3078858 | 2163032 | 14 | 98.99 | 0 | 2891 |
| *E. phoeniculicola* | II | GCA_000407505.1 | 3910972 | 889011 | 11 | 100 | 0 | 3507 |
| *E. villorum* | II | GCA_000407205.1 | 3060724 | 1302785 | 4 | 98.99 | 0 | 2821 |
| *E. avium* | III | GCA_000407245.1 | 4614282 | 652010 | 13 | 98.99 | 0 | 4504 |
| *E. gilvus* | III | GCA_000407545.1 | 4179913 | 3038269 | 5 | 98.99 | 0 | 4111 |
| *E. malodoratus* | III | GCA_000407185.1 | 4627134 | 1188036 | 7 | 98.99 | 0 | 4500 |
| *E. pallens* | III | GCA_000407485.1 | 5437454 | 1015307 | 13 | 100 | 0 | 5196 |
| *E. raffinosus* | III | GCA_000407525.1 | 4310366 | 1007288 | 9 | 98.99 | 0 | 4202 |
| *E. asini* | IV | GCA_000407365.1 | 2572706 | 2038454 | 2 | 98.99 | 0 | 2429 |
| *E. cecorum* | IV | GCA_000492155.1 | 2408477 | 1060610 | 6 | 98.99 | 0 | 2375 |
| *E. columbae* | IV | GCA_000407225.1 | 2545099 | 421089 | 9 | 98.99 | 0 | 2327 |
| *E. dispar* | IV | GCA_000407585.1 | 2812918 | 2801753 | 4 | 98.99 | 0 | 2635 |
| *E. saccharolyticus* | IV | GCA_000407285.1 | 2604038 | 2432614 | 2 | 98.99 | 0 | 2586 |
| *E. sulfureus* | IV | GCA_000407605.1 | 2301651 | 733717 | 8 | 98.99 | 0 | 2176 |

[1]*Enterococcus* clades, defined by (Lebreton et al., 2017).

[2]Number of contigs/scaffolds or chromosomes.

[3,4]Calculated using CheckM (Parks et al., 2015).

[5]Total CDS predicted by Prokka (Seemann, 2014).

**Supplementary Table. S7**. $F_{max}$ values for the full SwissProt dataset for the molecular function and cellular component ontologies.

| $F_{max}$ values for each of five species[1,2] | | | | | |
|---|---|---|---|---|---|
| **Method** | ***EC*** | ***MT*** | ***BS*** | ***PA*** | ***ST*** |
| **Molecular function** | | | | | |
| BLAST | 0.613 | 0.593 | 0.625 | 0.699 | 0.814 |
| Pfam | 0.650 | 0.549 | 0.571 | 0.534 | 0.559 |
| SAFPred-nn | 0.675 | 0.723 | 0.814 | 0.854 | 0.837 |
| SAFPred-synteny | 0.880 | **0.869** | **0.893** | **0.938** | 0.878 |
| SAFPred | **0.885** | **0.869** | **0.893** | **0.938** | 0.877 |
| DeepGOPlus | 0.686 | 0.755 | 0.841 | 0.883 | **0.911** |
| **Cellular component** | | | | | |
| BLAST | 0.569 | 0.397 | 0.638 | 0.700 | 0.871 |
| Pfam | 0.625 | 0.541 | 0.608 | 0.560 | 0.616 |
| SAFPred-nn | 0.731 | 0.500 | 0.898 | 0.900 | 0.917 |
| SAFPred-synteny | 0.920 | **0.847** | **0.943** | **0.945** | 0.918 |
| SAFPred | **0.922** | **0.847** | **0.943** | **0.945** | 0.917 |
| DeepGOPlus | 0.745 | 0.567 | 0.885 | 0.887 | **0.936** |

[1] EC: *Escherichia coli*, MT: *Mycobacterium tuberculosis*, BS: *Bacillus subtilis*, PA: *Pseudomonas aeruginosa* and ST: *Salmonella typhimurium*.

[2] Highest value for each species and for each GO category are shown in bold.

**Supplementary Table. S8.** $S_{min}$ values for the full SwissProt dataset.

| $S_{min}$ values for each of five species[1,2] | | | | | |
|---|---|---|---|---|---|
| **Method** | ***EC*** | ***MT*** | ***BS*** | ***PA*** | ***ST*** |
| **Biological process** | | | | | |
| BLAST | 20.97 | 22.93 | 15.99 | 19.02 | 8.53 |
| Pfam | 108.50 | 161.14 | 126.22 | 122.86 | 129.13 |
| SAFPred-nn (T5) | 18.61 | 18.44 | 9.84 | 12.76 | 8.83 |
| SAFPred-nn | 17.44 | 18.24 | 9.02 | 12.18 | 8.37 |
| SAFPred-synteny | **7.10** | **7.60** | **3.27** | **4.41** | 4.90 |
| SAFPred | **7.10** | **7.60** | **3.27** | **4.41** | 4.90 |
| DeepGOPlus | 16.45 | 14.94 | 5.68 | 10.39 | **2.85** |

| | | | | | |
|---|---|---|---|---|---|
| **Molecular function** | | | | | |
| BLAST | 10.97 | 11.07 | 7.99 | 8.25 | 5.51 |
| Pfam | 30.87 | 52.74 | 35.04 | 35.48 | 37.17 |
| SAFPred-nn (T5) | 10.01 | 8.99 | 4.74 | 4.52 | 5.72 |
| SAFPred-nn | 9.12 | 8.04 | 4.77 | 4.49 | 5.67 |
| SAFPred-synteny | **3.63** | **3.51** | **1.88** | **1.66** | 2.72 |
| SAFPred | **3.63** | **3.51** | **1.88** | **1.66** | 2.72 |
| DeepGOPlus | 8.68 | 6.36 | 2.57 | 2.67 | **1.68** |
| **Cellular component** | | | | | |
| BLAST | 6.46 | 5.74 | 3.87 | 4.17 | 2.53 |
| Pfam | 30.29 | 35.91 | 34.37 | 31.40 | 32.15 |
| SAFPred-nn (T5) | 4.87 | 4.99 | 1.76 | 1.81 | 2.37 |
| SAFPred-nn | 5.02 | 5.00 | 1.84 | 2.05 | 2.29 |
| SAFPred-synteny | **1.64** | **1.85** | **0.49** | **0.82** | 1.35 |
| SAFPred | **1.64** | **1.85** | **0.49** | **0.82** | 1.35 |
| DeepGOPlus | 4.92 | 4.63 | 1.55 | 1.77 | **1.18** |

[1] EC: *Escherichia coli*, MT: *Mycobacterium tuberculosis*, BS: *Bacillus subtilis*, PA: *Pseudomonas aeruginosa* and ST: *Salmonella typhimurium*.

[2] Lowest value for each species and for each GO category are shown in bold.

**Supplementary Table. S9.** Area under the precision/recall curve for the full SwissProt dataset, for all 5 bacterial organisms and three GO categories.

| Area under precision/recall curve for each of five species[1,2] | | | | | |
|---|---|---|---|---|---|
| **Method** | ***EC*** | ***MT*** | ***BS*** | ***PA*** | ***ST*** |
| **Biological process** | | | | | |
| BLAST | 0.519 | 0.497 | 0.686 | 0.642 | 0.642 |
| Pfam | 0.648 | 0.556 | 0.582 | 0.623 | 0.623 |
| SAFPred-nn (T5) | 0.529 | 0.514 | 0.76 | 0.714 | 0.714 |
| SAFPred-nn | 0.544 | 0.533 | 0.80 | 0.714 | 0.714 |
| DeepGOPlus | 0.564 | 0.600 | 0.862 | 0.797 | 0.797 |
| SAFPred-synteny | **0.855** | **0.805** | **0.907** | **0.908** | **0.908** |
| SAFPred | **0.855** | **0.805** | **0.907** | **0.908** | **0.908** |
| **Molecular function** | | | | | |
| BLAST | 0.569 | 0.573 | 0.658 | 0.644 | 0.644 |
| Pfam | 0.686 | 0.599 | 0.571 | 0.595 | 0.595 |
| SAFPred-nn (T5) | 0.523 | 0.602 | 0.764 | 0.774 | 0.774 |
| SAFPred-nn | 0.560 | 0.640 | 0.790 | 0.780 | 0.780 |
| DeepGOPlus | 0.613 | 0.719 | 0.857 | 0.899 | 0.899 |
| SAFPred-synteny | **0.872** | **0.849** | **0.887** | **0.921** | **0.921** |
| SAFPred | **0.872** | **0.849** | **0.887** | **0.921** | **0.921** |
| **Cellular component** | | | | | |
| BLAST | 0.541 | 0.324 | 0.701 | 0.724 | 0.724 |
| Pfam | 0.675 | 0.598 | 0.608 | 0.634 | 0.634 |
| SAFPred-nn (T5) | 0.641 | 0.344 | 0.858 | 0.849 | 0.849 |
| SAFPred-nn | 0.633 | 0.335 | 0.879 | 0.853 | 0.853 |
| DeepGOPlus | 0.747 | 0.512 | 0.931 | 0.917 | 0.917 |
| SAFPred-synteny | **0.919** | **0.831** | **0.954** | **0.945** | **0.945** |
| SAFPred | **0.919** | **0.831** | **0.954** | **0.945** | **0.945** |

[1] EC: *Escherichia coli*, MT: *Mycobacterium tuberculosis*, BS: *Bacillus subtilis*, PA: *Pseudomonas aeruginosa* and ST: *Salmonella typhimurium*

[2] The highest value for each species and for each GO category are shown in bold

**Supplementary Table. S10.** $F_{max}$ values for the entire *E. coli* SwissProt benchmark sets.

| Method | 40 | 50 | 60 | 70 | 80 | Full |
|---|---|---|---|---|---|---|
| **Biological process** | | | | | | |
| BLAST | 0.505 | 0.526 | 0.540 | 0.556 | 0.560 | 0.570 |
| Pfam | 0.610 | 0.610 | 0.610 | 0.610 | 0.610 | 0.610 |
| SAFPred-nn (T5) | 0.464 | 0.510 | 0.574 | 0.596 | 0.607 | 0.625 |
| SAFPred-nn | 0.536 | 0.584 | 0.623 | 0.635 | 0.639 | 0.646 |
| DeepGOPlus | 0.568 | 0.601 | 0.624 | 0.636 | 0.644 | 0.648 |
| SAFPred-synteny | 0.720 | 0.792 | 0.824 | 0.840 | 0.858 | 0.877 |
| SAFPred | 0.724 | 0.800 | 0.830 | 0.846 | 0.865 | 0.877 |
| **Molecular function** | | | | | | |
| BLAST | 0.554 | 0.573 | 0.585 | 0.599 | 0.603 | 0.613 |
| Pfam | 0.650 | 0.650 | 0.650 | 0.650 | 0.650 | 0.650 |
| SAFPred-nn (T5) | 0.475 | 0.528 | 0.595 | 0.611 | 0.618 | 0.632 |
| SAFPred-nn | 0.558 | 0.598 | 0.656 | 0.666 | 0.669 | 0.675 |
| DeepGOPlus | 0.605 | 0.641 | 0.649 | 0.674 | 0.680 | 0.686 |
| SAFPred-synteny | 0.725 | 0.803 | 0.833 | 0.850 | 0.865 | 0.886 |
| SAFPred | 0.739 | 0.810 | 0.840 | 0.855 | 0.872 | 0.886 |
| **Cellular component** | | | | | | |
| BLAST | 0.502 | 0.519 | 0.532 | 0.546 | 0.554 | 0.569 |
| Pfam | 0.625 | 0.625 | 0.625 | 0.625 | 0.625 | 0.625 |
| SAFPred-nn (T5) | 0.560 | 0.610 | 0.691 | 0.712 | 0.732 | 0.738 |
| SAFPred-nn | 0.610 | 0.650 | 0.707 | 0.715 | 0.723 | 0.731 |
| DeepGOPlus | 0.645 | 0.705 | 0.721 | 0.706 | 0.726 | 0.745 |
| SAFPred-synteny | 0.769 | 0.835 | 0.866 | 0.887 | 0.905 | 0.925 |
| SAFPred | 0.774 | 0.842 | 0.873 | 0.893 | 0.911 | 0.925 |

**Supplementary Table. S11.** $F_{max}$ values for the entire *M. tuberculosis* SwissProt benchmark sets.

| Method | 40 | 50 | 60 | 70 | 80 | Full |
|---|---|---|---|---|---|---|
| **Biological process** | | | | | | |
| BLAST | 0.525 | 0.521 | 0.531 | 0.532 | 0.539 | 0.543 |
| Pfam | 0.513 | 0.513 | 0.513 | 0.513 | 0.513 | 0.513 |
| SAFPred-nn (T5) | 0.520 | 0.597 | 0.613 | 0.615 | 0.618 | 0.618 |
| SAFPred-nn | 0.575 | 0.617 | 0.629 | 0.630 | 0.633 | 0.636 |
| DeepGOPlus | 0.627 | 0.645 | 0.670 | 0.667 | 0.667 | 0.669 |
| SAFPred-synteny | 0.698 | 0.750 | 0.786 | 0.812 | 0.825 | 0.838 |
| SAFPred | 0.703 | 0.750 | 0.787 | 0.813 | 0.828 | 0.838 |
| **Molecular function** | | | | | | |
| BLAST | 0.589 | 0.582 | 0.580 | 0.584 | 0.591 | 0.593 |
| Pfam | 0.549 | 0.549 | 0.549 | 0.549 | 0.549 | 0.549 |
| SAFPred-nn (T5) | 0.586 | 0.654 | 0.672 | 0.677 | 0.683 | 0.681 |
| SAFPred-nn | 0.654 | 0.706 | 0.714 | 0.718 | 0.722 | 0.723 |
| DeepGOPlus | 0.709 | 0.736 | 0.745 | 0.752 | 0.750 | 0.755 |
| SAFPred-synteny | 0.748 | 0.800 | 0.830 | 0.851 | 0.864 | 0.869 |
| SAFPred | 0.748 | 0.801 | 0.832 | 0.856 | 0.866 | 0.869 |
| **Cellular component** | | | | | | |
| BLAST | 0.401 | 0.397 | 0.394 | 0.396 | 0.396 | 0.397 |
| Pfam | 0.541 | 0.541 | 0.541 | 0.541 | 0.541 | 0.541 |
| SAFPred-nn (T5) | 0.434 | 0.513 | 0.520 | 0.517 | 0.510 | 0.507 |
| SAFPred-nn | 0.431 | 0.504 | 0.505 | 0.505 | 0.502 | 0.500 |
| DeepGOPlus | 0.570 | 0.577 | 0.575 | 0.573 | 0.572 | 0.567 |
| SAFPred-synteny | 0.634 | 0.700 | 0.753 | 0.804 | 0.835 | 0.846 |
| SAFPred | 0.638 | 0.704 | 0.756 | 0.807 | 0.835 | 0.846 |

**Supplementary Table. S12.** $F_{max}$ values for the entire *B. subtilis* SwissProt benchmark sets.

| Method | 40 | 50 | 60 | 70 | 80 | Full |
|---|---|---|---|---|---|---|
| **Biological process** | | | | | | |
| BLAST | 0.628 | 0.636 | 0.634 | 0.638 | 0.638 | 0.639 |
| Pfam | 0.582 | 0.582 | 0.582 | 0.582 | 0.582 | 0.582 |
| SAFPred-nn (T5) | 0.700 | 0.768 | 0.783 | 0.788 | 0.790 | 0.791 |
| SAFPred-nn | 0.752 | 0.809 | 0.821 | 0.826 | 0.827 | 0.828 |
| DeepGOPlus | 0.791 | 0.835 | 0.841 | 0.852 | 0.857 | 0.857 |
| SAFPred-synteny | 0.822 | 0.881 | 0.897 | 0.907 | 0.913 | 0.921 |
| SAFPred | 0.828 | 0.885 | 0.900 | 0.909 | 0.914 | 0.921 |
| **Molecular function** | | | | | | |
| BLAST | 0.622 | 0.630 | 0.623 | 0.624 | 0.625 | 0.625 |
| Pfam | 0.571 | 0.571 | 0.571 | 0.571 | 0.571 | 0.571 |
| SAFPred-nn (T5) | 0.672 | 0.758 | 0.782 | 0.789 | 0.791 | 0.791 |
| SAFPred-nn | 0.735 | 0.789 | 0.805 | 0.809 | 0.813 | 0.814 |
| DeepGOPlus | 0.775 | 0.830 | 0.836 | 0.842 | 0.850 | 0.841 |
| SAFPred-synteny | 0.798 | 0.860 | 0.878 | 0.888 | 0.891 | 0.898 |
| SAFPred | 0.803 | 0.864 | 0.884 | 0.892 | 0.893 | 0.898 |
| **Cellular component** | | | | | | |
| BLAST | 0.622 | 0.623 | 0.631 | 0.637 | 0.638 | 0.638 |
| Pfam | 0.608 | 0.608 | 0.608 | 0.608 | 0.608 | 0.608 |
| SAFPred-nn (T5) | 0.766 | 0.859 | 0.874 | 0.883 | 0.883 | 0.884 |
| SAFPred-nn | 0.811 | 0.870 | 0.888 | 0.896 | 0.897 | 0.898 |
| DeepGOPlus | 0.820 | 0.848 | 0.863 | 0.888 | 0.864 | 0.885 |
| SAFPred-synteny | 0.878 | 0.920 | 0.926 | 0.935 | 0.941 | 0.944 |
| SAFPred | 0.874 | 0.925 | 0.929 | 0.939 | 0.941 | 0.944 |

**Supplementary Table. S13.** $F_{max}$ values for the entire *P. aeruginosa* SwissProt benchmark sets.

| Method | 40 | 50 | 60 | 70 | 80 | Full |
|---|---|---|---|---|---|---|
| **Biological process** | | | | | | |
| BLAST | 0.650 | 0.666 | 0.680 | 0.686 | 0.684 | 0.683 |
| Pfam | 0.579 | 0.579 | 0.579 | 0.579 | 0.579 | 0.579 |
| SAFPred-nn (T5) | 0.629 | 0.731 | 0.786 | 0.798 | 0.797 | 0.796 |
| SAFPred-nn | 0.681 | 0.769 | 0.794 | 0.799 | 0.797 | 0.797 |
| DeepGOPlus | 0.749 | 0.785 | 0.812 | 0.820 | 0.816 | 0.824 |
| SAFPred-synteny | 0.754 | 0.835 | 0.879 | 0.892 | 0.916 | 0.927 |
| SAFPred | 0.755 | 0.837 | 0.878 | 0.896 | 0.922 | 0.927 |
| **Molecular function** | | | | | | |
| BLAST | 0.712 | 0.721 | 0.716 | 0.708 | 0.702 | 0.699 |
| Pfam | 0.534 | 0.534 | 0.534 | 0.534 | 0.534 | 0.534 |
| SAFPred-nn (T5) | 0.679 | 0.801 | 0.848 | 0.858 | 0.856 | 0.853 |
| SAFPred-nn | 0.765 | 0.849 | 0.863 | 0.863 | 0.857 | 0.854 |
| DeepGOPlus | 0.810 | 0.856 | 0.883 | 0.894 | 0.882 | 0.883 |
| SAFPred-synteny | 0.789 | 0.879 | 0.913 | 0.921 | 0.932 | 0.938 |
| SAFPred | 0.784 | 0.882 | 0.915 | 0.925 | 0.939 | 0.938 |
| **Cellular component** | | | | | | |
| BLAST | 0.658 | 0.687 | 0.692 | 0.701 | 0.701 | 0.700 |
| Pfam | 0.560 | 0.560 | 0.560 | 0.560 | 0.560 | 0.560 |
| SAFPred-nn (T5) | 0.743 | 0.838 | 0.876 | 0.894 | 0.900 | 0.898 |
| SAFPred-nn | 0.780 | 0.868 | 0.894 | 0.900 | 0.900 | 0.900 |
| DeepGOPlus | 0.819 | 0.847 | 0.874 | 0.877 | 0.900 | 0.887 |
| SAFPred-synteny | 0.823 | 0.890 | 0.914 | 0.927 | 0.934 | 0.945 |
| SAFPred | 0.830 | 0.893 | 0.914 | 0.931 | 0.943 | 0.945 |

**Supplementary Table. S14.** $F_{max}$ values for the entire *S. typhimurium* SwissProt benchmark sets.

| Method | 40 | 50 | 60 | 70 | 80 | Full |
|---|---|---|---|---|---|---|
| **Biological process** | | | | | | |
| BLAST | 0.675 | 0.728 | 0.775 | 0.800 | 0.822 | 0.852 |
| Pfam | 0.579 | 0.579 | 0.579 | 0.579 | 0.579 | 0.579 |
| SAFPred-nn (T5) | 0.634 | 0.739 | 0.795 | 0.848 | 0.875 | 0.869 |
| SAFPred-nn | 0.774 | 0.855 | 0.889 | 0.910 | 0.919 | 0.880 |
| DeepGOPlus | 0.811 | 0.860 | 0.896 | 0.911 | 0.922 | 0.928 |
| SAFPred-synteny | 0.811 | 0.871 | 0.894 | 0.906 | 0.908 | 0.905 |
| SAFPred | 0.811 | 0.871 | 0.895 | 0.907 | 0.910 | 0.905 |
| **Molecular function** | | | | | | |
| BLAST | 0.674 | 0.723 | 0.751 | 0.782 | 0.811 | 0.814 |
| Pfam | 0.559 | 0.559 | 0.559 | 0.559 | 0.559 | 0.559 |
| SAFPred-nn (T5) | 0.589 | 0.694 | 0.753 | 0.818 | 0.856 | 0.829 |
| SAFPred-nn | 0.761 | 0.844 | 0.868 | 0.894 | 0.904 | 0.837 |
| DeepGOPlus | 0.800 | 0.845 | 0.875 | 0.892 | 0.904 | 0.911 |
| SAFPred-synteny | 0.796 | 0.863 | 0.884 | 0.893 | 0.889 | 0.883 |
| SAFPred | 0.798 | 0.862 | 0.885 | 0.895 | 0.890 | 0.883 |
| **Cellular component** | | | | | | |
| BLAST | 0.644 | 0.705 | 0.737 | 0.774 | 0.815 | 0.871 |
| Pfam | 0.616 | 0.616 | 0.616 | 0.616 | 0.616 | 0.616 |
| SAFPred-nn (T5) | 0.794 | 0.848 | 0.871 | 0.898 | 0.918 | 0.916 |
| SAFPred-nn | 0.824 | 0.881 | 0.909 | 0.923 | 0.941 | 0.917 |
| DeepGOPlus | 0.819 | 0.868 | 0.898 | 0.901 | 0.917 | 0.936 |
| SAFPred-synteny | 0.856 | 0.896 | 0.912 | 0.920 | 0.922 | 0.918 |

| | | | | | | |
|---|---|---|---|---|---|---|
| SAFPred | 0.858 | 0.899 | 0.916 | 0.922 | 0.923 | 0.918 |

**Supplementary Table. S15.** Prediction coverage[1] (in %) for the full SwissProt dataset.

| Prediction coverage for each of five species (%)[2] | | | | | |
|---|---|---|---|---|---|
| **Method** | ***EC*** | ***MT*** | ***BS*** | ***PA*** | ***ST*** |
| **Biological process** | | | | | |
| BLAST | 65.66 | 66.14 | 75.58 | 73.11 | 87.61 |
| Pfam | 81.41 | 71.19 | 80.69 | 75.03 | 84.42 |
| SAFPred-nn (T5) | 84.78 | 91.40 | 91.58 | 91.00 | 97.15 |
| SAFPred-nn | 88.29 | 90.16 | 94.88 | 94.12 | 97.82 |
| DeepGOPlus | 88.79 | 81.92 | 94.80 | 95.80 | 97.49 |
| SAFPred-synteny | 92.48 | 82.36 | 90.68 | 92.32 | 90.45 |
| SAFPred | 92.66 | 82.36 | 90.68 | 92.32 | 90.45 |
| **Molecular function** | | | | | |
| BLAST | 74.04 | 69.51 | 75.25 | 81.36 | 89.23 |
| Pfam | 88.46 | 88.80 | 87.28 | 88.82 | 87.44 |
| SAFPred-nn (T5) | 89.64 | 96.53 | 91.47 | 98.59 | 97.06 |
| SAFPred-nn | 88.35 | 96.00 | 93.56 | 98.33 | 97.23 |
| DeepGOPlus | 98.05 | 92.80 | 89.69 | 94.86 | 96.25 |
| SAFPred-synteny | 92.85 | 92.98 | 88.29 | 93.06 | 88.42 |
| SAFPred | 93.33 | 92.98 | 88.29 | 93.06 | 88.42 |
| **Cellular component** | | | | | |
| BLAST | 60.29 | 58.41 | 68.377 | 77.51 | 87.05 |
| Pfam | 81.65 | 76.35 | 81.981 | 84.43 | 89.68 |
| SAFPred-nn (T5) | 82.39 | 67.22 | 93.437 | 93.60 | 96.15 |
| SAFPred-nn | 81.36 | 64.92 | 94.153 | 93.43 | 96.76 |
| DeepGOPlus | 89.86 | 95.08 | 97.017 | 94.29 | 97.98 |

| | | | | | |
|---|---|---|---|---|---|
| SAFPred-synteny | 93.32 | 92.86 | 95.943 | 91.35 | 96.96 |
| SAFPred | 93.53 | 92.86 | 95.943 | 91.35 | 96.96 |

[1] The number of test proteins annotated with at least one GO term at the threshold which maximizes the F1-score.
[2] EC: *Escherichia coli*, MT: *Mycobacterium tuberculosis*, BS: *Bacillus subtilis*, PA: *Pseudomonas aeruginosa* and ST: *Salmonella typhimurium*

**Supplementary Table. S16.** Prediction coverage[1] (in %) for the entire SwissProt benchmark evaluation, averaged over 5 bacterial organisms for three GO categories. The standard deviation across the 5 bacterial organisms is indicated in parentheses.

| Method | Maximum sequence % ID between the training and the test set | | | | | |
|---|---|---|---|---|---|---|
| | 40% | 50% | 60% | 70% | 80% | Full |
| **Biological process** | | | | | | |
| BLAST | 71.16% (3.30%) | 70.20% (5.82%) | 70.21% (5.00%) | 71.76% (5.62%) | 72.02% (4.49%) | 73.62% (7.99%) |
| Pfam[2] | 78.55% (4.77%) | 78.55% (4.77%) | 78.55% (4.77%) | 78.55% (4.77%) | 78.55% (4.77%) | 78.55% (4.77%) |
| SAFPred-nn (T5) | 85.71% (4.99%) | 88.23% (6.43%) | 90.09% (3.74%) | 89.03% (4.80%) | 89.93% (4.49%) | 91.18% (3.92%) |
| SAFPred-nn | 85.33% (4.07%) | 91.58% (6.72%) | 93.23% (3.52%) | 92.40% (4.24%) | 92.76% (3.78%) | 93.06% (3.41%) |
| DeepGOPlus | 95.62% (1.56%) | 94.48% (3.45%) | 93.84% (4.99%) | 94.44% (3.16%) | 90.25% (6.16%) | 91.76% (5.73%) |
| SAFPred-synteny | 85.48% (3.18%) | 87.36% (1.05%) | 87.93% (3.90%) | 89.95% (4.43%) | 91.42% (0.83%) | 89.66% (3.74%) |
| SAFPred | 84.04% (1.82%) | 87.93% (0.92%) | 88.11% (3.90%) | 89.17% (3.60%) | 91.73% (0.91%) | 89.69% (3.77%) |
| **Molecular function** | | | | | | |
| BLAST | 75.42% (3.39%) | 75.52% (2.93%) | 74.33% (3.73%) | 74.51% (4.34%) | 75.17% (4.20%) | 77.88% (6.82%) |
| Pfam[2] | 88.16% (0.67%) | 88.16% (0.67%) | 88.16% (0.67%) | 88.16% (0.67%) | 88.16% (0.67%) | 88.16% (0.67%) |
| SAFPred-nn (T5) | 89.55% (4.20%) | 92.16% (6.19%) | 94.44% (3.49%) | 94.46% (3.41%) | 94.62% (3.10%) | 94.66% (3.47%) |
| SAFPred-nn | 89.67% (5.32%) | 92.59% (6.82%) | 94.83% (3.31%) | 94.68% (3.32%) | 94.69% (3.49%) | 94.69% (3.55%) |
| DeepGOPlus | 93.79% (2.32%) | 93.98% (3.29%) | 94.46% (2.71%) | 93.98% (3.00%) | 95.25% (2.48%) | 94.33% (2.89%) |
| SAFPred-synteny | 87.07% (3.14%) | 88.57% (1.60%) | 90.65% (1.65%) | 91.35% (1.97%) | 91.36% (2.33%) | 91.12% (2.26%) |
| SAFPred | 85.37% (2.80%) | 89.20% (1.75%) | 90.96% (1.49%) | 91.69% (2.02%) | 91.79% (2.50%) | 91.21% (2.34%) |
| **Cellular component** | | | | | | |
| BLAST | 71.81% (8.69%) | 67.46% (5.39%) | 68.98% (7.05%) | 70.76% (7.77%) | 67.96% (7.00%) | 70.33% (10.75%) |
| Pfam[2] | 82.82% (4.33%) | 82.82% (4.33%) | 82.82% (4.33%) | 82.82% (4.33%) | 82.82% (4.33%) | 82.82% (4.33%) |
| SAFPred-nn (T5) | 71.44% (12.05%) | 82.58% (11.93%) | 86.49% (8.77%) | 86.49% (9.561%) | 86.47% (9.99%) | 86.56% (10.77%) |
| SAFPred-nn | 74.37% (12.89%) | 83.43% (12.24%) | 86.28% (11.03%) | 86.31% (11.50%) | 86.56% (11.99%) | 86.12% (11.86%) |
| DeepGOPlus | 94.66% (1.63%) | 95.86% (1.52%) | 93.86% (1.86%) | 95.23% (1.89%) | 94.88% (2.90%) | 94.84% (2.82%) |

| | | | | | | |
|---|---|---|---|---|---|---|
| SAFPred-synteny | 85.93% (4.14%) | 91.17% (3.14%) | 90.54% (5.40%) | 91.42% (3.94%) | 93.50% (2.48%) | 94.09% (2.07%) |
| SAFPred | 85.87% (5.42%) | 91.01% (3.82%) | 92.08% (3.44%) | 92.91% (4.08%) | 93.89% (2.17%) | 94.13% (2.05%) |

[1] The number of test proteins annotated with at least one GO term at the threshold which maximizes the F1-score.
[2] Because the Pfam method uses a different training set for predictions, it is not possible to compare the change in the coverage across different train/test pairs.

**Supplementary Table S17.** Maximum % sequence identity of *Enterococcus* toxin genes to the 11 putative novel toxins predicted by SAFPred.

| epx gene type | epx gene locus tag | predicted gene locus tag | maximum identity (%) |
|---|---|---|---|
| epx1 | GCA_015300525.1_02780 | Ente_haem_BAA-382_V2_01251 | 38.89 |
| epx1 | GCA_002945555.1_00127 | Ente_haem_BAA-382_V2_01826 | 25.76 |
| epx1 | GCA_003319525.1_02660 | Ente_haem_BAA-382_V2_01826 | 25.76 |
| epx1 | GCA_004120285.1_01193 | Ente_haem_BAA-382_V2_01826 | 25.76 |
| epx1 | GCA_004125665.1_01702 | Ente_haem_BAA-382_V2_01826 | 25.76 |
| epx1 | GCA_004125915.1_01060 | Ente_haem_BAA-382_V2_01826 | 25.76 |
| epx1 | GCA_004125935.1_01411 | Ente_haem_BAA-382_V2_01826 | 25.76 |
| epx1 | GCA_004125945.1_01477 | Ente_haem_BAA-382_V2_01826 | 25.76 |
| epx1 | GCA_004125955.1_00929 | Ente_haem_BAA-382_V2_01826 | 25.76 |
| epx1 | GCA_004126025.1_01270 | Ente_haem_BAA-382_V2_01826 | 25.76 |
| epx1 | GCA_005236595.1_02731 | Ente_haem_BAA-382_V2_01826 | 25.76 |
| epx1 | GCA_005237765.1_02590 | Ente_haem_BAA-382_V2_01826 | 25.76 |
| epx1 | GCA_005238535.1_01041 | Ente_haem_BAA-382_V2_01826 | 25.76 |
| epx1 | GCA_015302065.1_02752 | Ente_haem_BAA-382_V2_01826 | 25.76 |
| epx1 | GCA_015333445.1_02601 | Ente_haem_BAA-382_V2_01826 | 25.76 |
| epx1 | GCA_015335045.1_02694 | Ente_haem_BAA-382_V2_01826 | 25.76 |
| epx1 | GCA_015336125.1_02821 | Ente_haem_BAA-382_V2_01826 | 25.76 |
| epx1 | GCA_015338255.1_02588 | Ente_haem_BAA-382_V2_01826 | 25.76 |

| | | | |
|---|---|---|---|
| epx4 | GCA_017356565.1_01452 | Ente_mund_ATCC882_V5_00469 | 60.00 |
| epx4 | GCA_015506475.1_02489 | Ente_haem_BAA-382_V2_01251 | 28.85 |
| epx4 | GCA_015507715.1_02409 | Ente_haem_BAA-382_V2_01251 | 28.85 |
| epx4 | GCA_018089265.1_02226 | Ente_haem_BAA-382_V2_01251 | 28.85 |

# Supplementary Figures

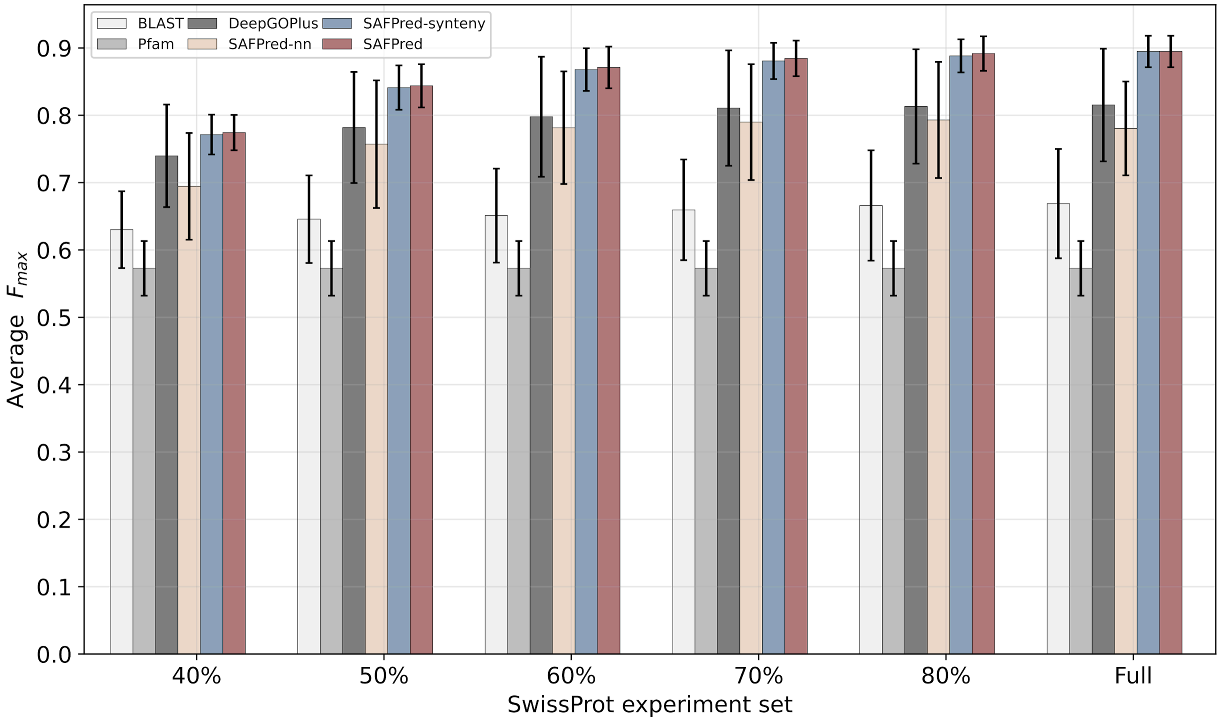


**Supplementary Fig. S4.** average $F_{max}$ values in Molecular Function Ontology (MFO), across benchmarking datasets for five bacteria in our experimental setup, and the error bars show the corresponding standard deviation of each method, across five species. Note that bar plots for the Pfam baseline are identical for all 6 experiment sets because Pfam uses a different training set, independent of our experimental design.

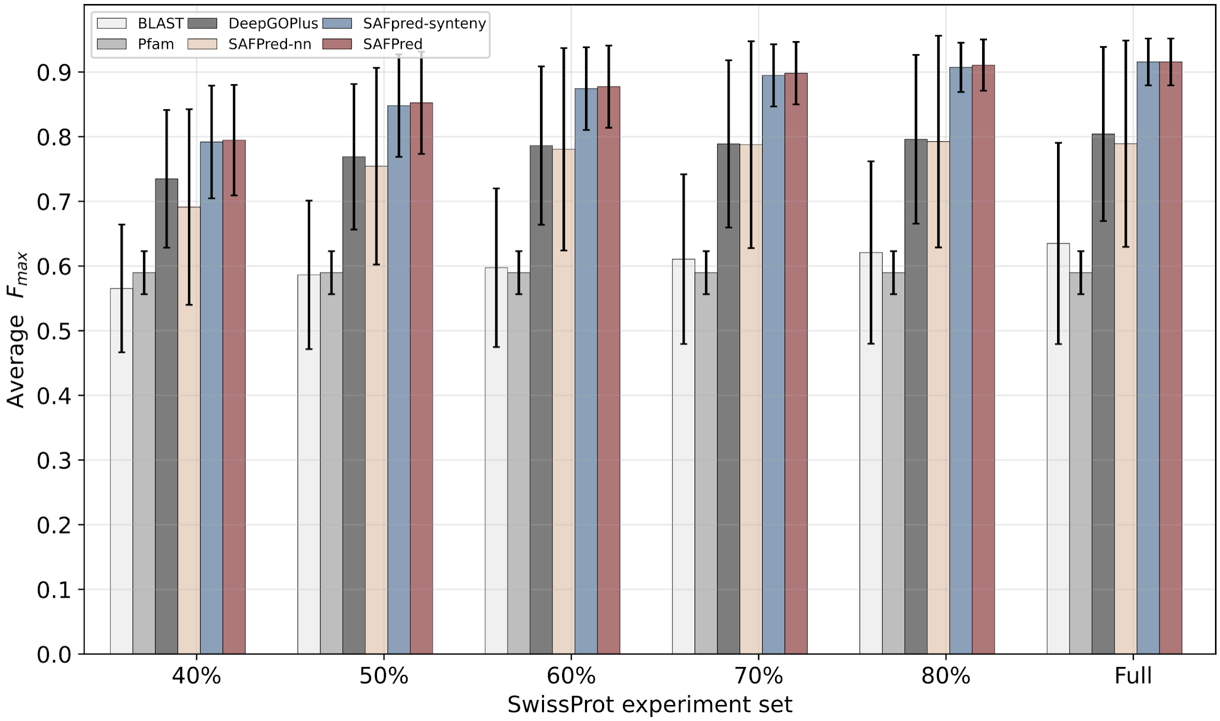


**Supplementary Fig. S5.** average $F_{max}$ values in Cellular Component Ontology (CCO), across benchmarking datasets for five bacteria in our experimental setup, and the error bars show the corresponding standard deviation of each method, across five species. Note that bar plots for the Pfam baseline are identical for all 6 experiment sets because Pfam uses a different training set, independent of our experimental design.

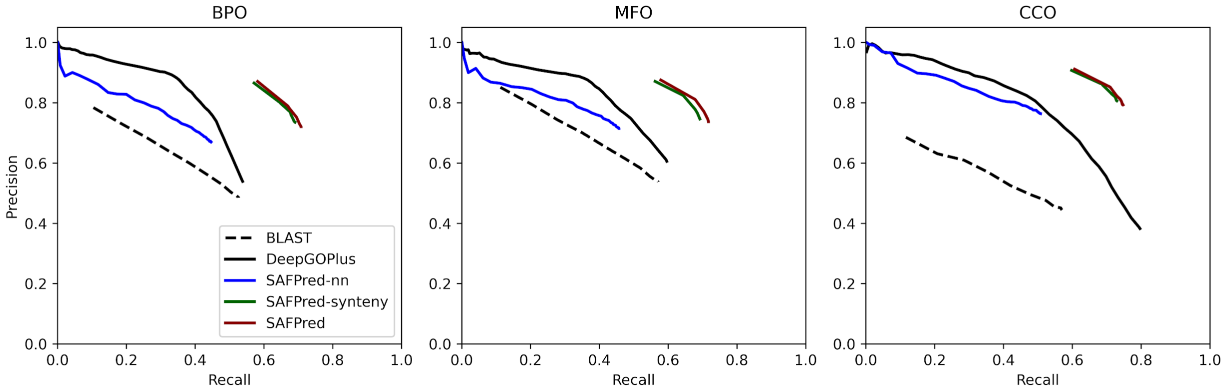


**Supplementary Fig. S6.** Precision recall curves for the E. coli dataset where the % sequence ID between the training and the test is limited to 40%.

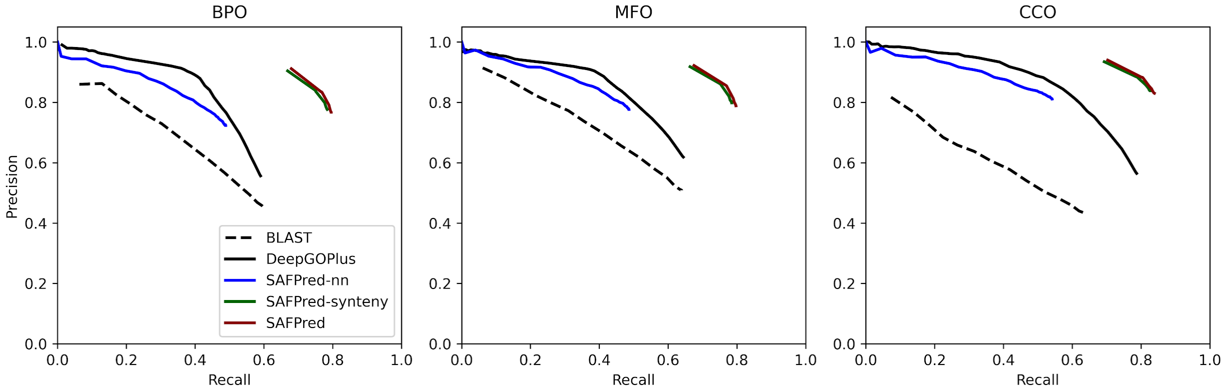


**Supplementary Fig. S7.** Precision recall curves for the E. coli dataset where the % sequence ID between the training and the test is limited to 50%.

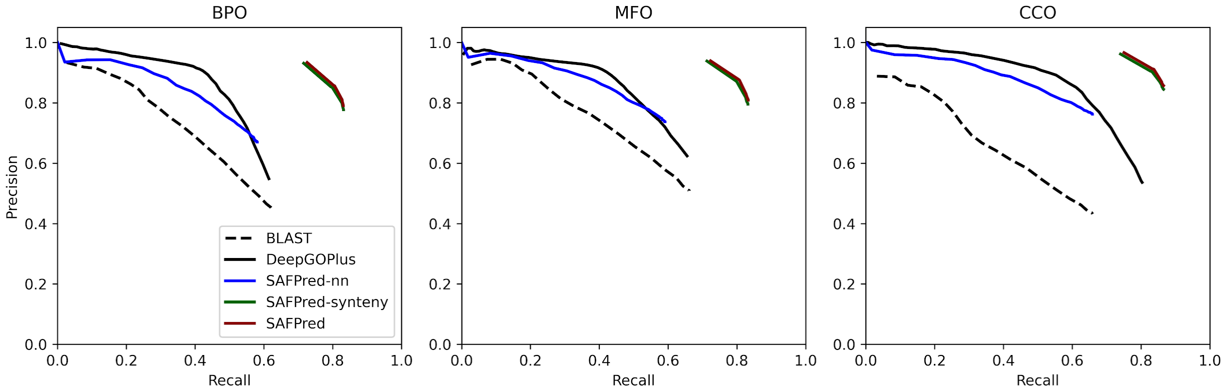


**Supplementary Fig. S8.** Precision recall curves for the E. coli dataset where the % sequence ID between the training and the test is limited to 60%.

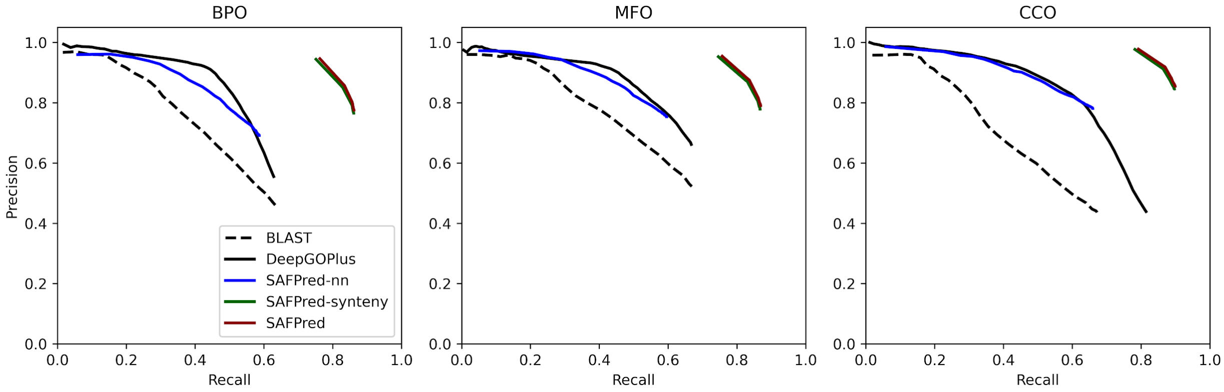


**Supplementary Fig. S9.** Precision recall curves for the E. coli dataset where the % sequence ID between the training and the test is limited to 70%.

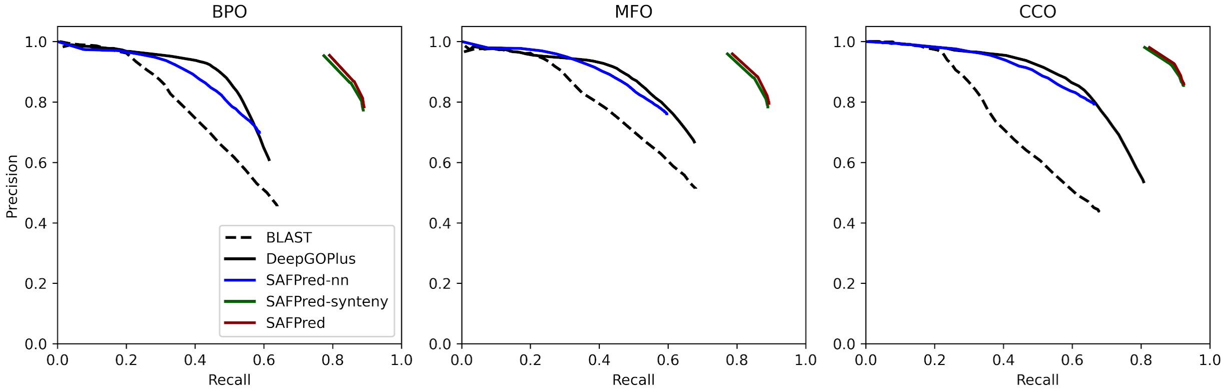


**Supplementary Fig. S10.** Precision recall curves for the E. coli dataset where the % sequence ID between the training and the test is limited to 80%.

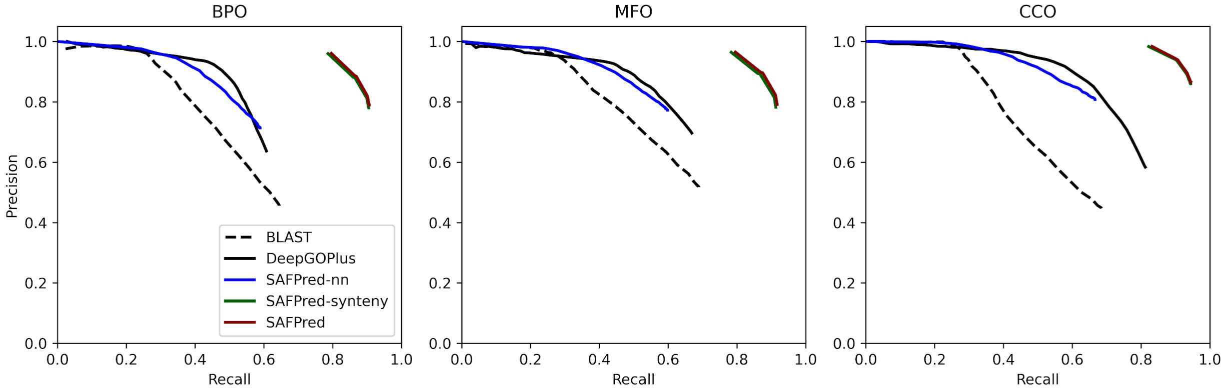


**Supplementary Fig. S11.** Precision recall curves for the E. coli dataset where the % sequence ID between the training and the test is limited to 95% (full dataset).

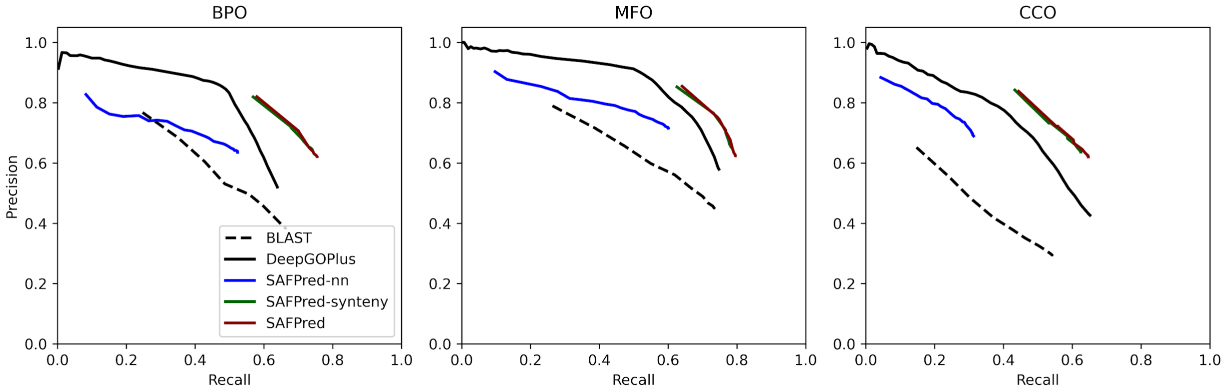


**Supplementary Fig. S12.** Precision recall curves for the *M. tuberculosis* dataset where the % sequence ID between the training and the test is limited to 40%.

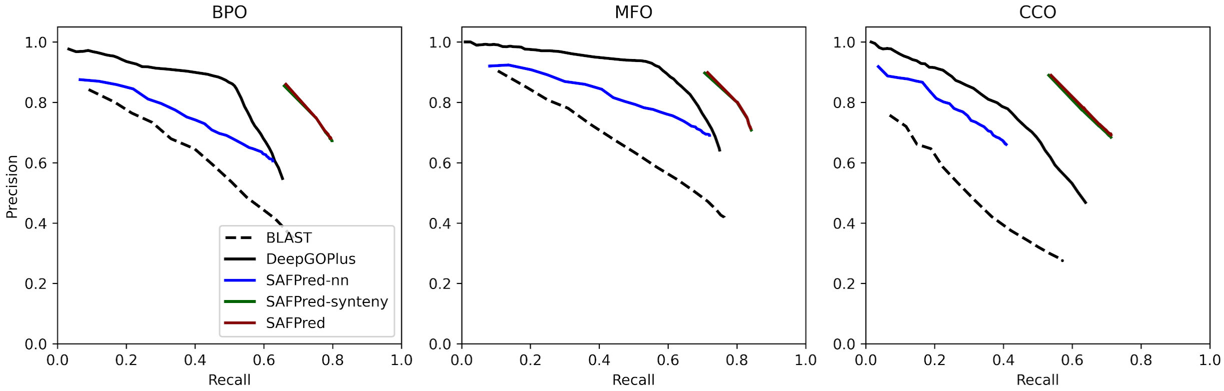


**Supplementary Fig. S13.** Precision recall curves for the *M. tuberculosis* dataset where the % sequence ID between the training and the test is limited to 50%.

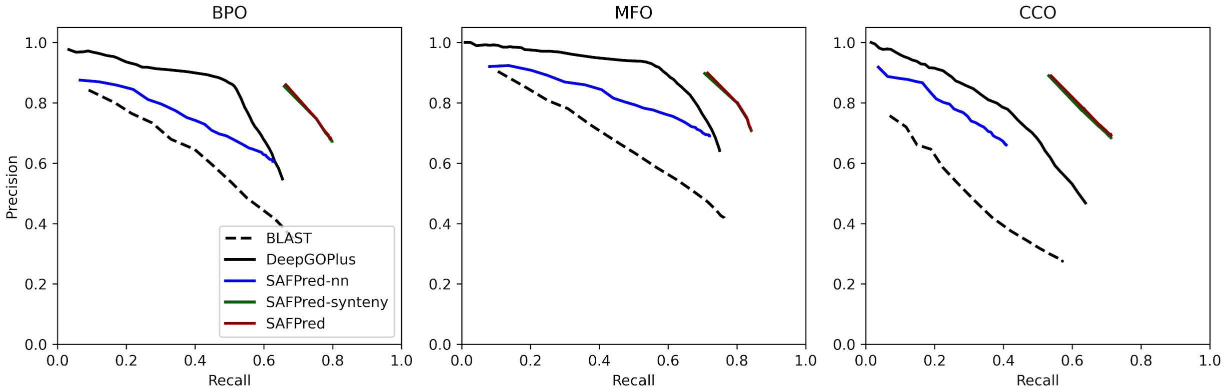


**Supplementary Fig. S14.** Precision recall curves for the *M. tuberculosis* dataset where the % sequence ID between the training and the test is limited to 60%.

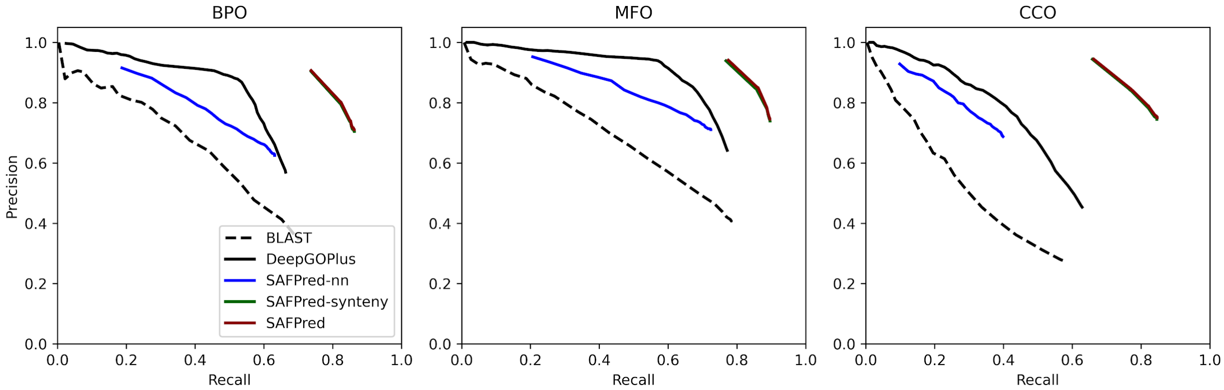


**Supplementary Fig. S15.** Precision recall curves for the *M. tuberculosis* dataset where the % sequence ID between the training and the test is limited to 70%.

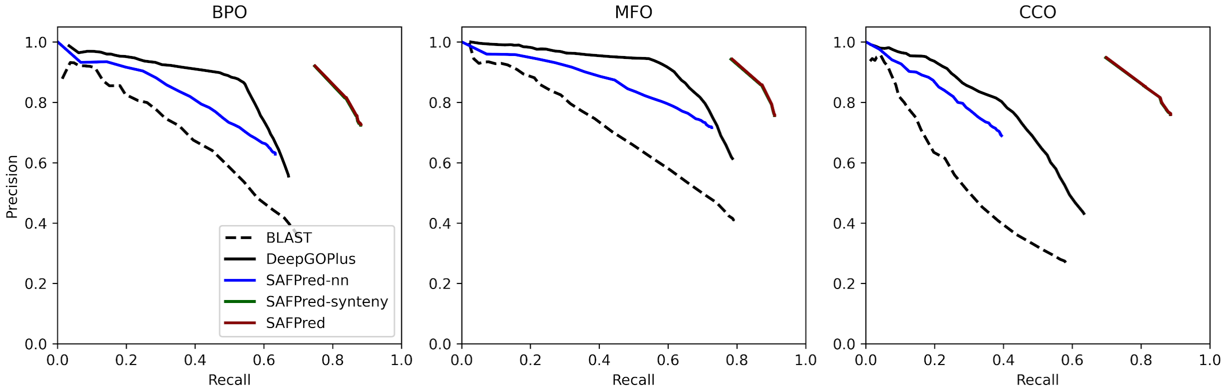


**Supplementary Fig. S16.** Precision recall curves for the *M. tuberculosis* dataset where the % sequence ID between the training and the test is limited to 80%.

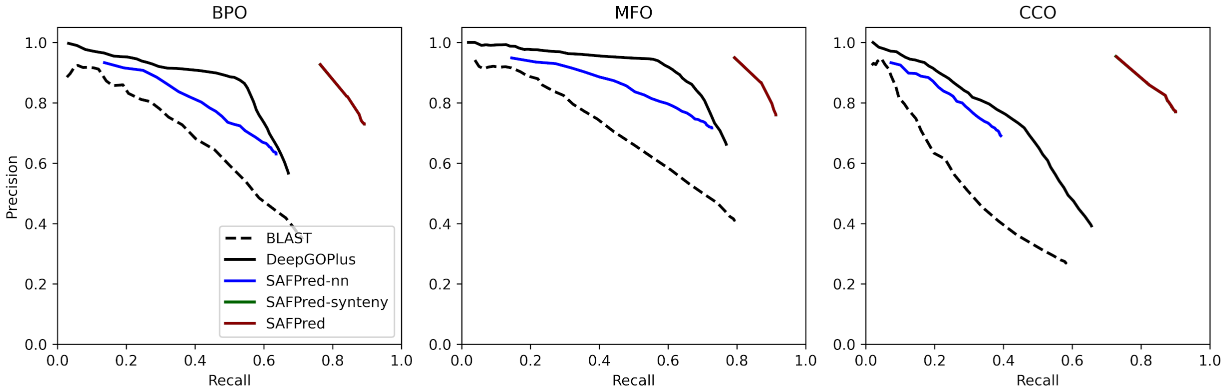


**Supplementary Fig. S17.** Precision recall curves for the *M. tuberculosis* dataset where the % sequence ID between the training and the test is limited to 95% (full dataset).

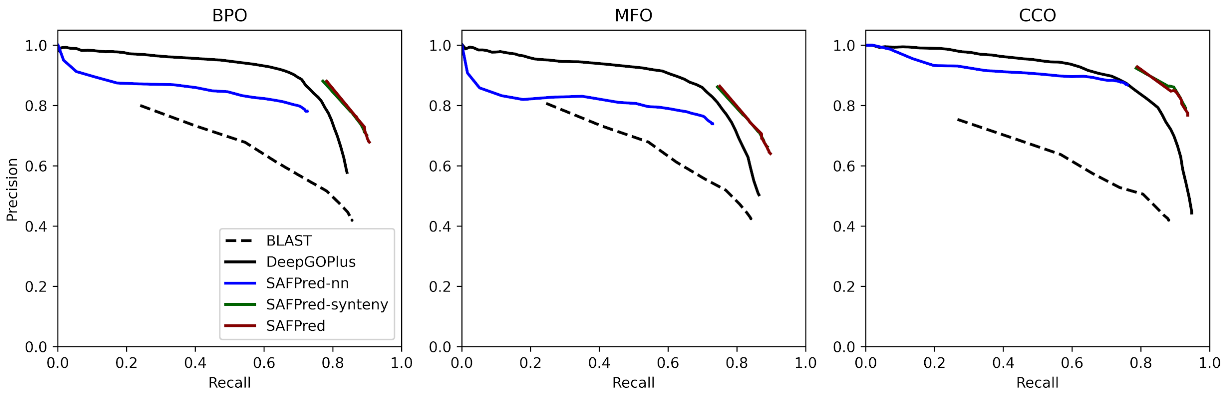


**Supplementary Fig. S18.** Precision recall curves for the *B. subtilis* dataset where the % sequence ID between the training and the test is limited to 40%.

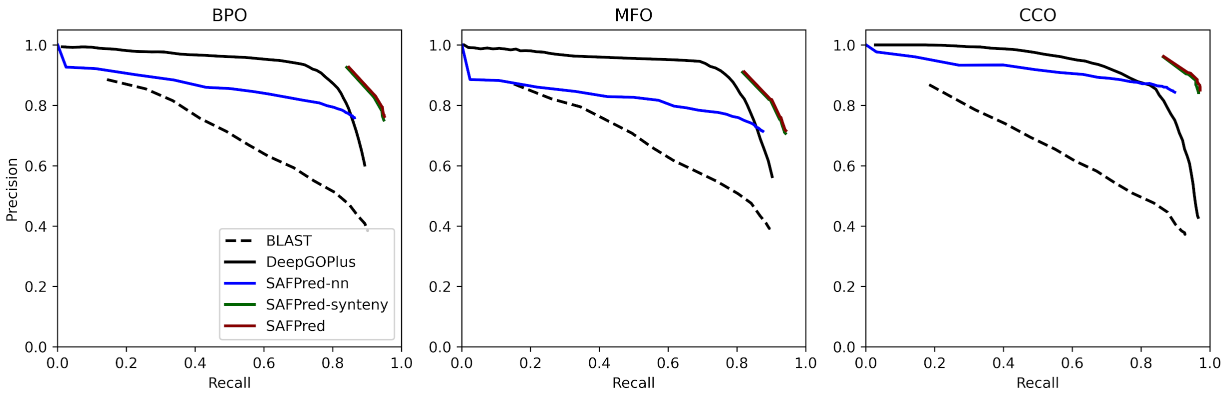


**Supplementary Fig. S19.** Precision recall curves for the *B. subtilis* dataset where the % sequence ID between the training and the test is limited to 50%.

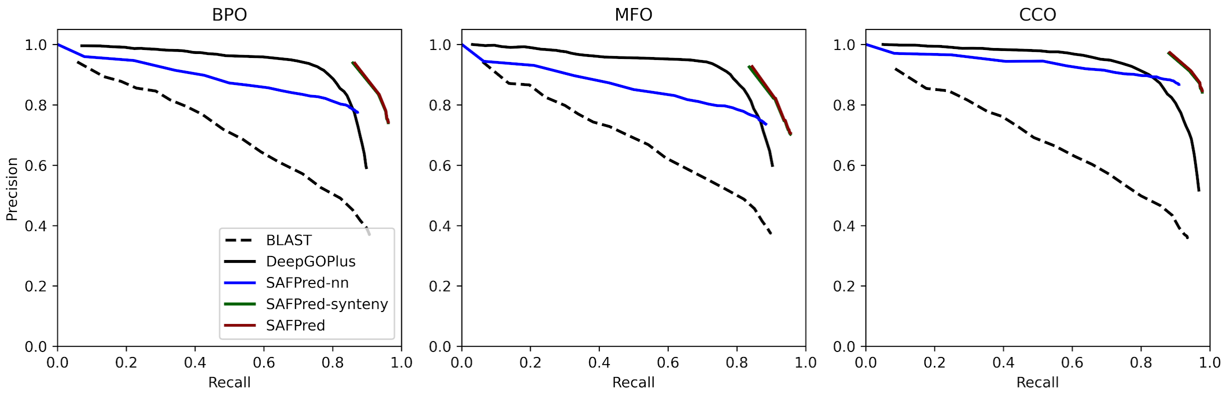


**Supplementary Fig. S20.** Precision recall curves for the *B. subtilis* dataset where the % sequence ID between the training and the test is limited to 60%.

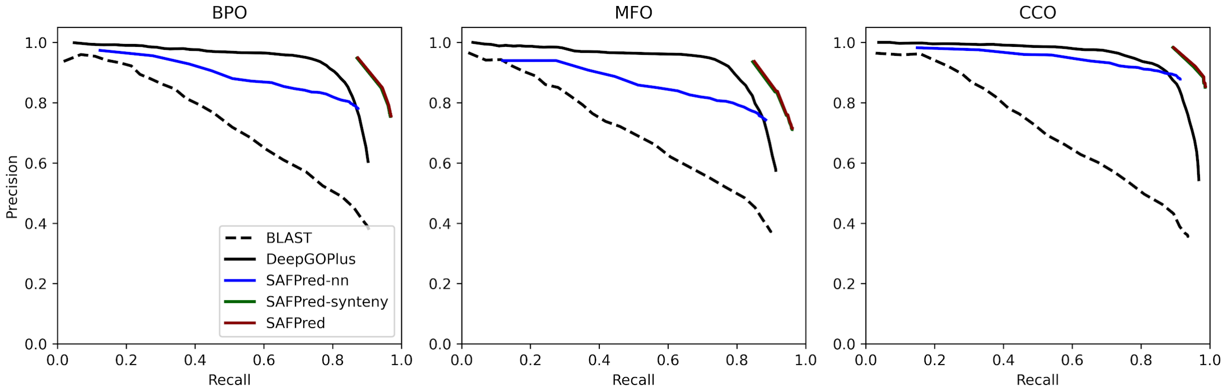


**Supplementary Fig. S21.** Precision recall curves for the *B. subtilis* dataset where the % sequence ID between the training and the test is limited to 70%.

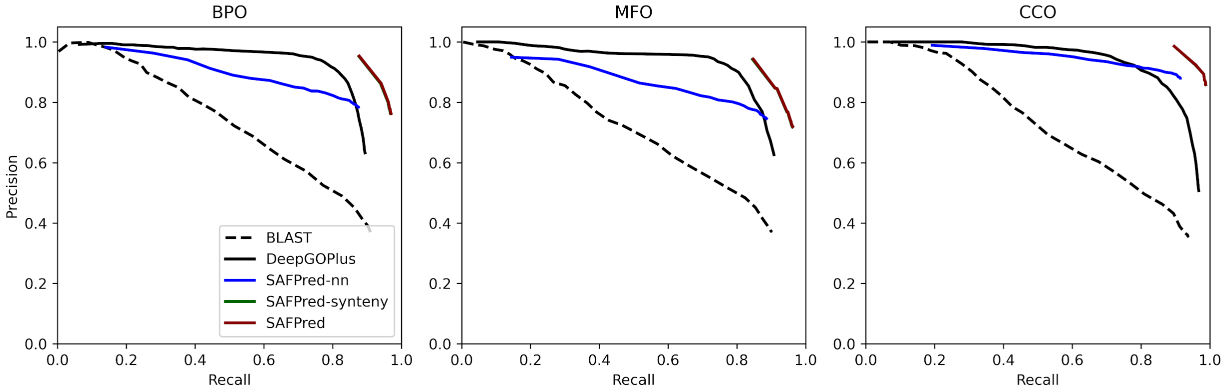


**Supplementary Fig. S22.** Precision recall curves for the *B. subtilis* dataset where the % sequence ID between the training and the test is limited to 80%.

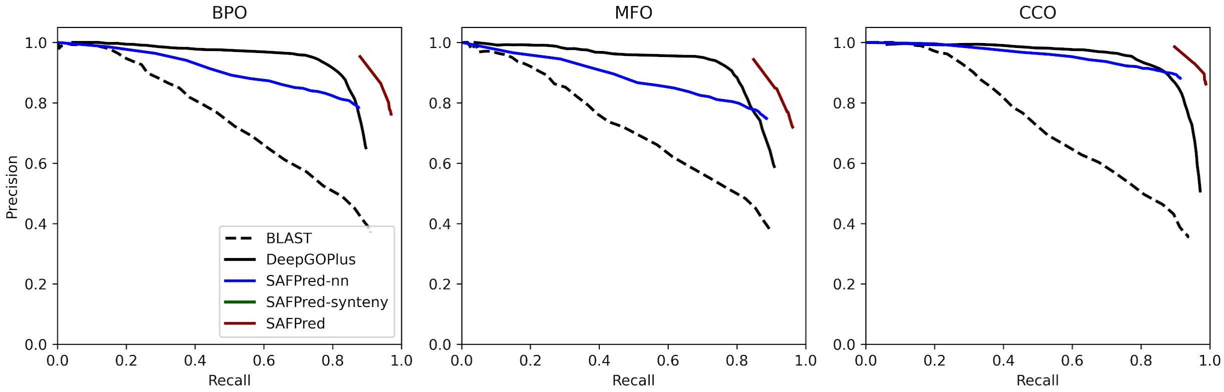


**Supplementary Fig. S23.** Precision recall curves for the *B. subtilis* dataset where the % sequence ID between the training and the test is limited to 95% (full dataset).

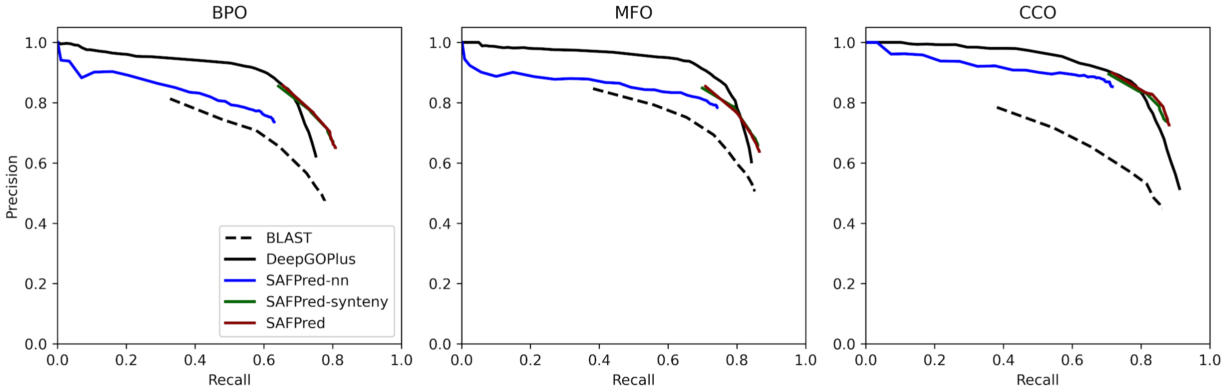


**Supplementary Fig. S24.** Precision recall curves for the *P. aeruginosa* dataset where the % sequence ID between the training and the test is limited to 40%.

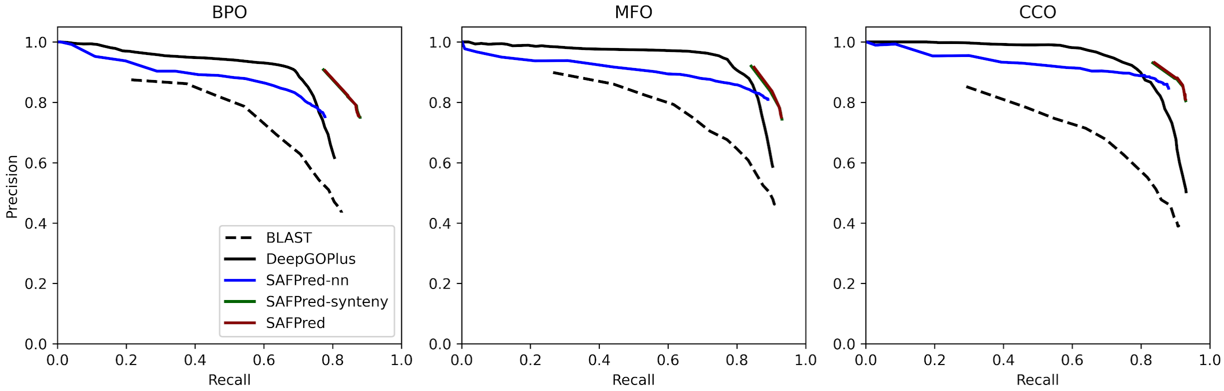


**Supplementary Fig. S25.** Precision recall curves for the *P. aeruginosa* dataset where the % sequence ID between the training and the test is limited to 50%.

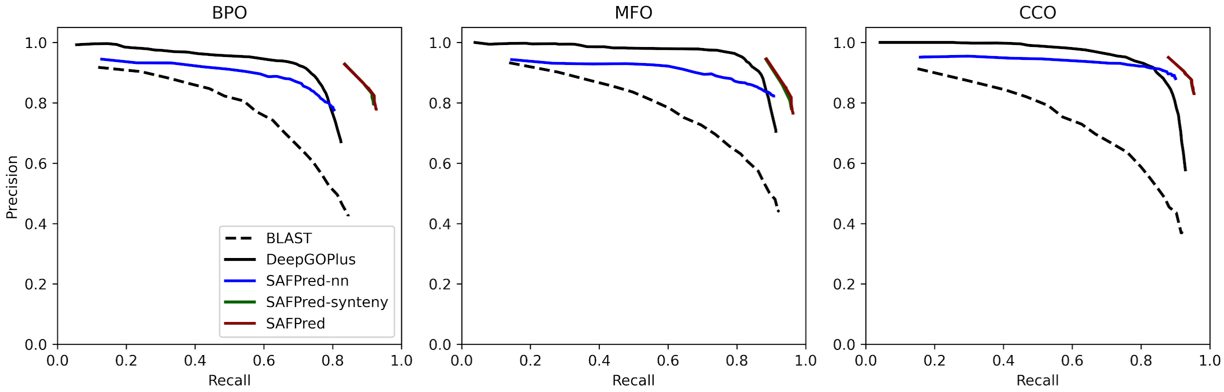


**Supplementary Fig. S26.** Precision recall curves for the *P. aeruginosa* dataset where the % sequence ID between the training and the test is limited to 60%.

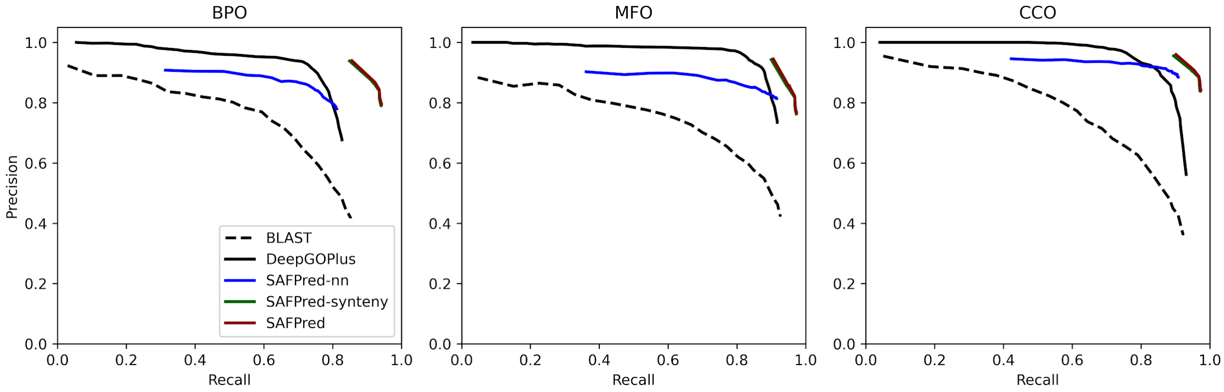


**Supplementary Fig. S27.** Precision recall curves for the *P. aeruginosa* dataset where the % sequence ID between the training and the test is limited to 70%.

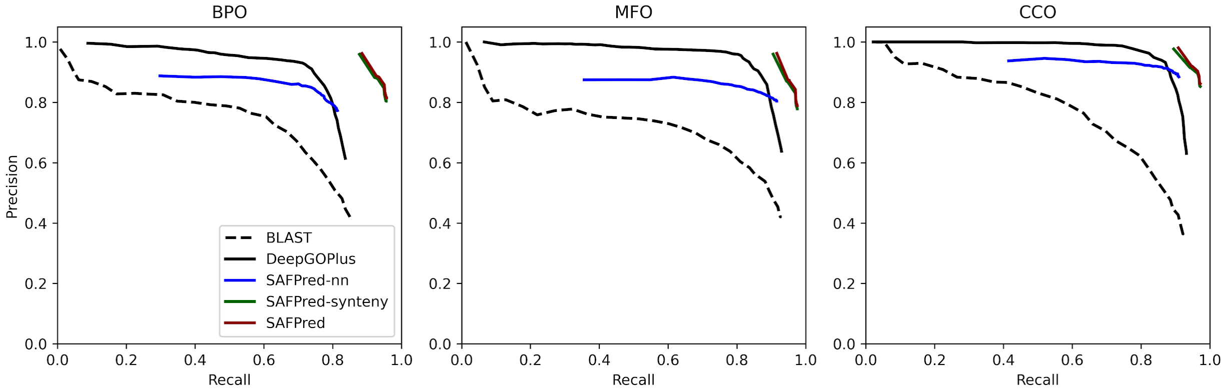


**Supplementary Fig. S28.** Precision recall curves for the *P. aeruginosa* dataset where the % sequence ID between the training and the test is limited to 80%.

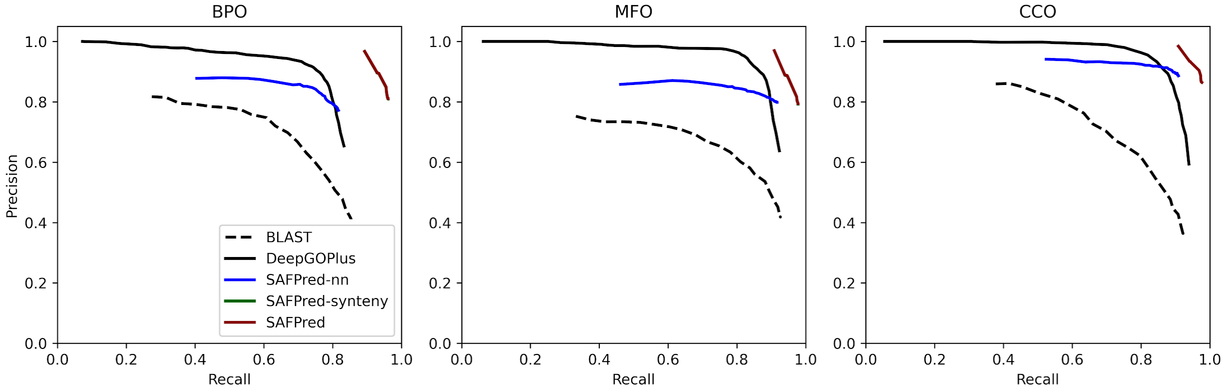


**Supplementary Fig. S29.** Precision recall curves for the *P. aeruginosa* dataset where the % sequence ID between the training and the test is limited to 95% (full dataset).

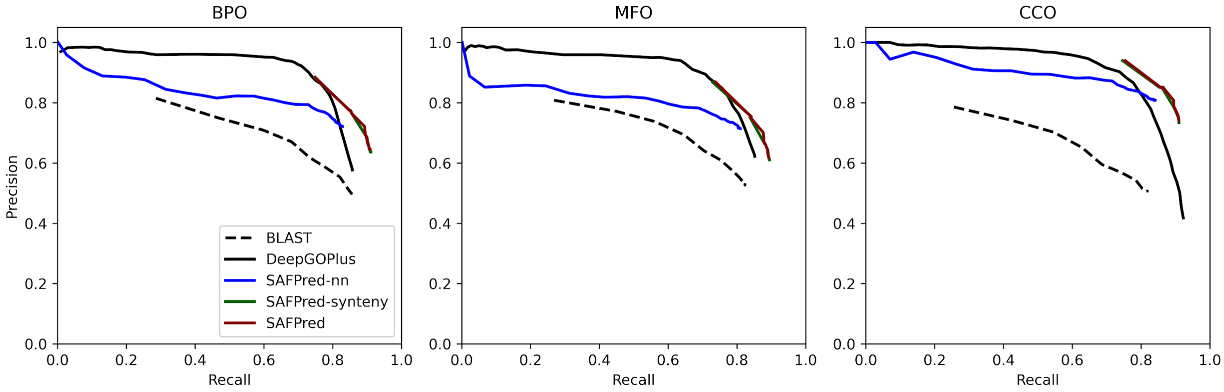


**Supplementary Fig. S30.** Precision recall curves for the *S. typhimurium* dataset where the % sequence ID between the training and the test is limited to 40%.

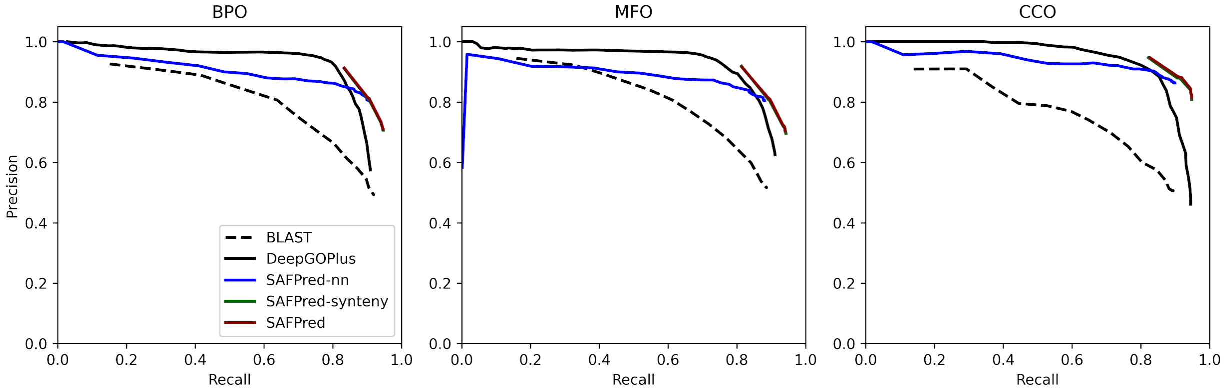


**Supplementary Fig. S31.** Precision recall curves for the *S. typhimurium* dataset where the % sequence ID between the training and the test is limited to 50%.

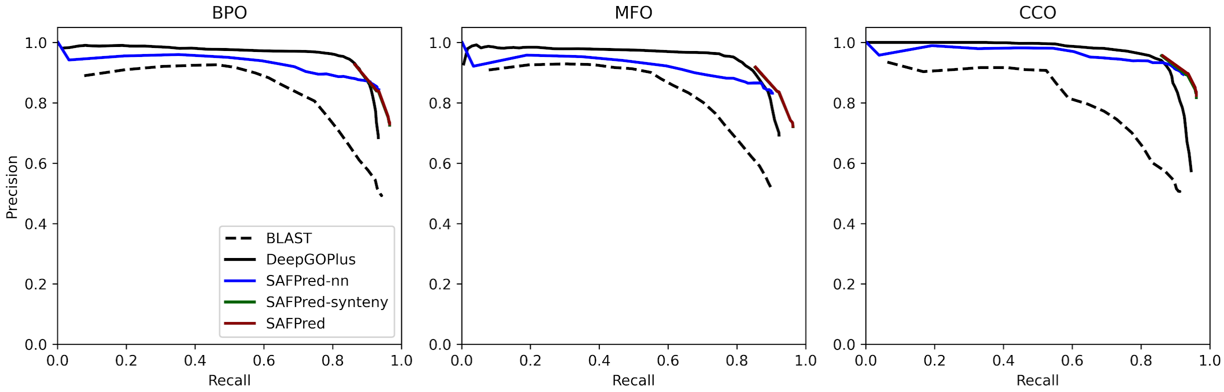


**Supplementary Fig. S32.** Precision recall curves for the *S. typhimurium* dataset where the % sequence ID between the training and the test is limited to 60%.

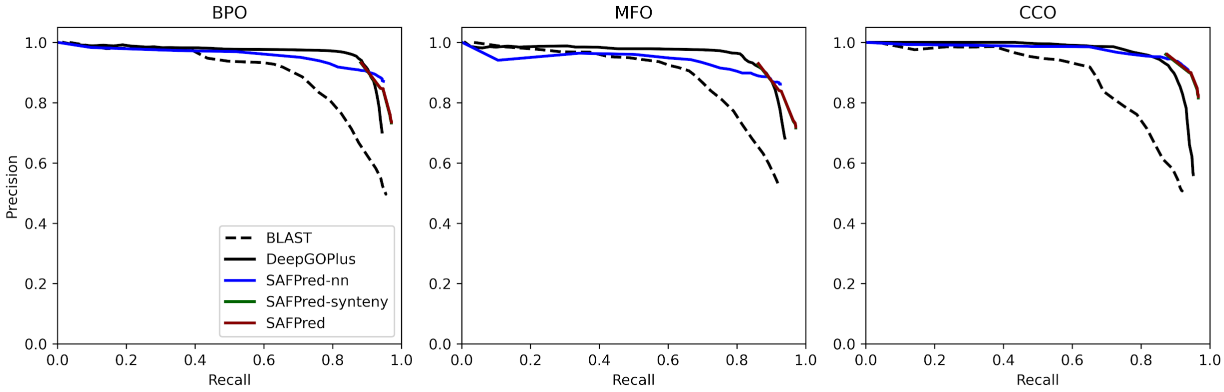


**Supplementary Fig. S33.** Precision recall curves for the *S. typhimurium* dataset where the % sequence ID between the training and the test is limited to 70%.

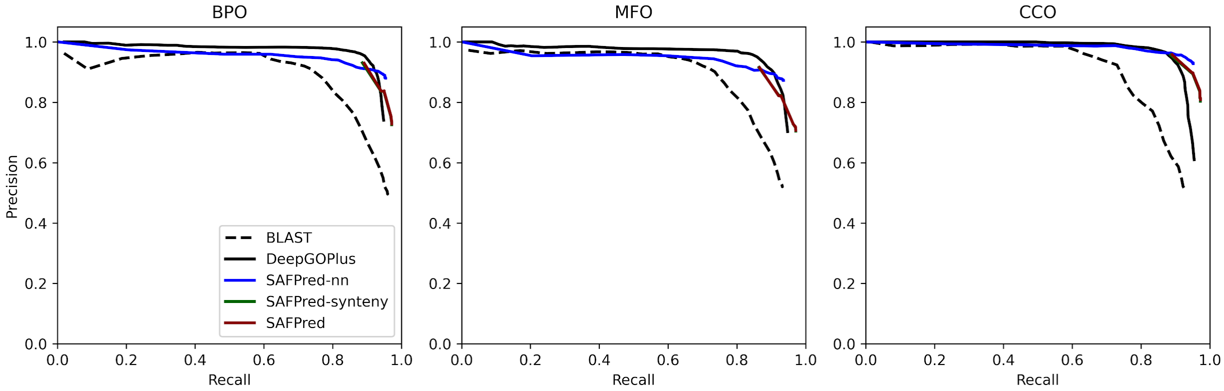


**Supplementary Fig. S34.** Precision recall curves for the *S. typhimurium* dataset where the % sequence ID between the training and the test is limited to 80%.

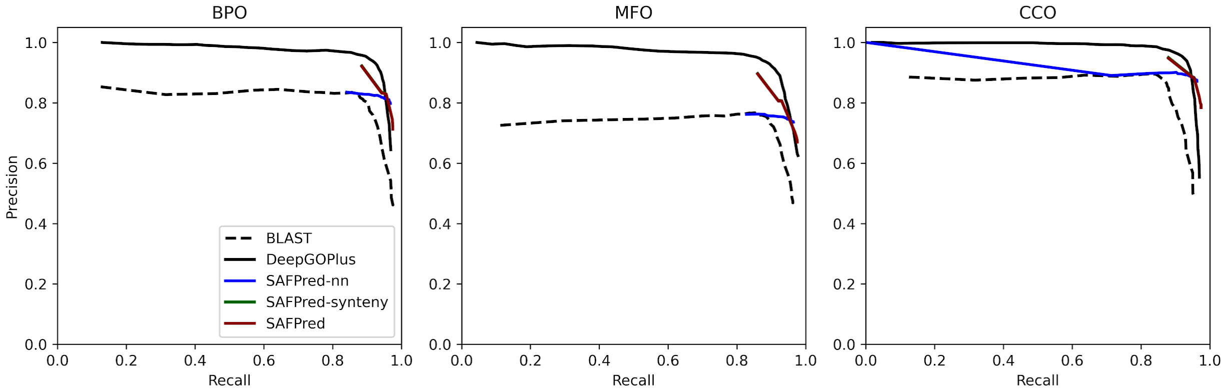


**Supplementary Fig. S35.** Precision recall curves for the *S. typhimurium* dataset where the % sequence ID between the training and the test is limited to 95% (full dataset).

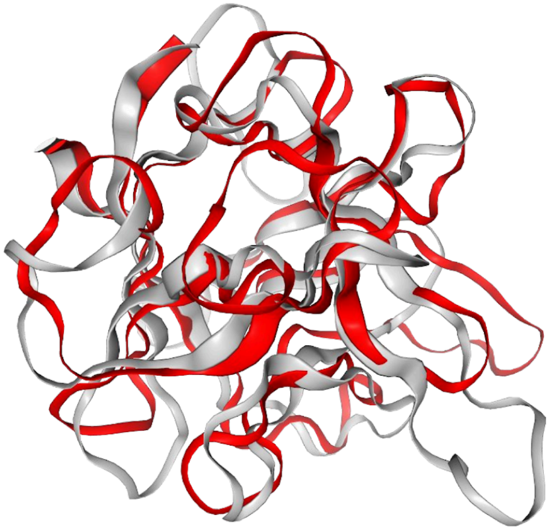

Sequence ID: 0.058
TM-Score: 0.76

```
Query
E. haemoperoxidus BAA-382 0174
38 NRNYVIKANPEGFKAKYWKTRYVD----VGSDWVNLTTDKDN-GSSYQLVTNGIVQENVAYKIYDSGSG-KYLNYQAGNT
          +   +++                    +     V+    VN   +     +     + L                +       +    + L   +A

Target
epx1
1 MAHVTLQSLS--NNDLCLDVYGENGDKTVAGGSVNGWSCHGSWNQVWGLDK--------EERYRSRVASDRCLTVNAD--
```

**Supplementary Fig. S36.** SAFPred predicts a possible new variant of pore-forming toxin gene in *E. haemoperoxidus*, distantly related to the known toxin Epx1, as evidenced by the highly significant structural alignment to the known toxin Epx1. Gene 0174 (red ribbon, query) had a TM-alignment score of 0.76 when we aligned its structure to Epx1 with Foldseek (gray ribbon, target), despite low sequence identity (5.8%). The protein's sequence alignment is shown below the 3D structural alignment.

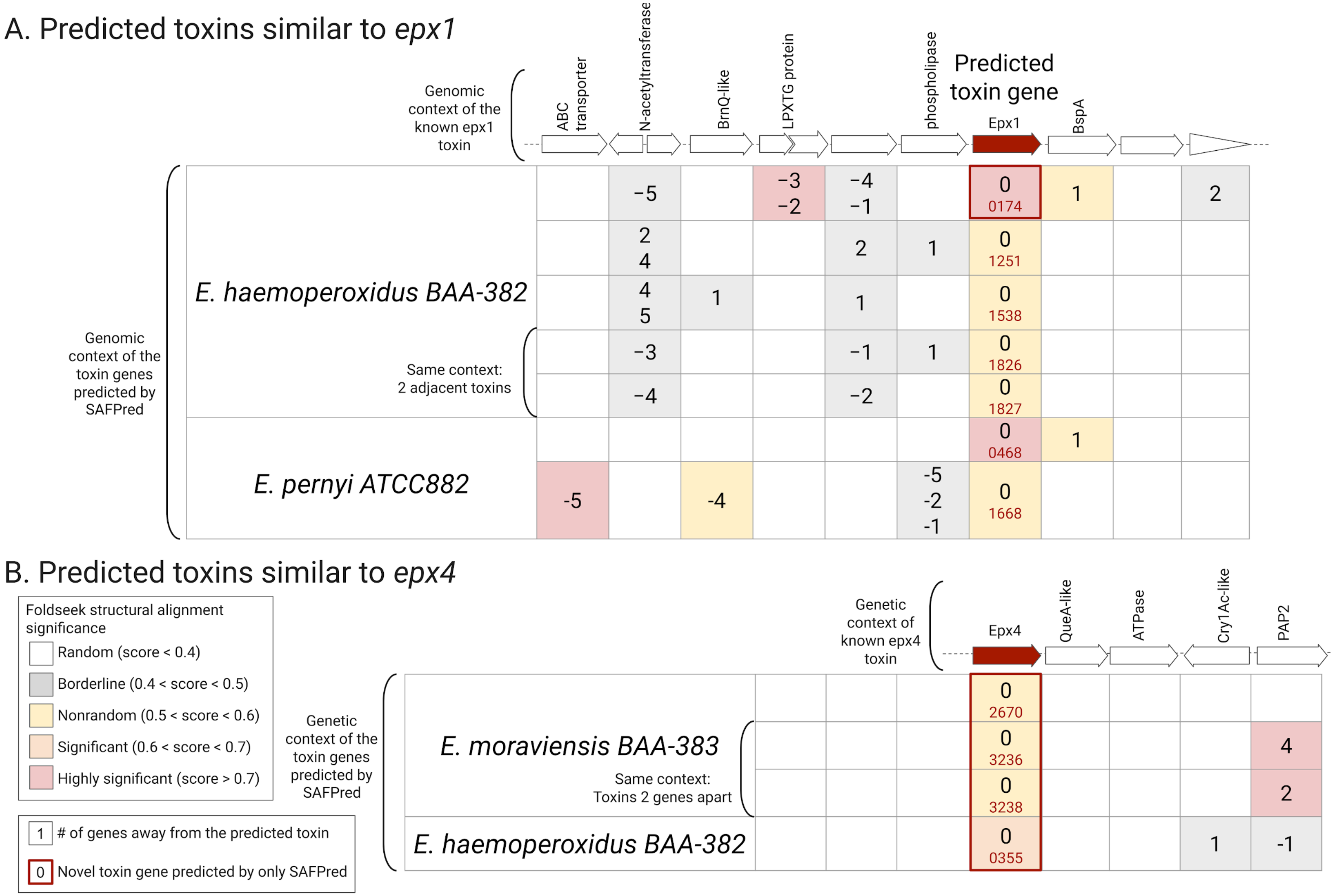


**Supplementary Fig. S37.** SAFPred predicts 11 likely novel toxin genes in our *Enterococcus* dataset (aligned below the column titled "Predicted toxin gene"), including five that could not be predicted by previous function prediction methods (red frames). A) seven genes with the highest similarity to *E. faecalis epx1*. These genes also shared some similarity in genomic context with *epx1*. B) four genes with the highest similarity to *E. hirae epx4*. These genes also shared some similarity in genomic context with *epx4*. The operon diagrams show the known genomic contexts of *epx1* and *exp4* found in *E. faecalis* and *E. hirae* species that were studied, respectively (Xiong et al., 2022). Beneath this, cells in the table represent the occurrence of genes with structural similarity to those in the known *epx1* or *epx4* operons. Their relative position within the operon in reference to the predicted toxin gene (# of genes away from the predicted toxin) and structural alignment score (coloring) obtained using Foldseek to the analogous gene in the operon diagram is shown. Gene locus tags, a 4-digit number given based on their location within the genome, for the predicted toxins are also placed within the cells. Additional details are shown in Figure S8.

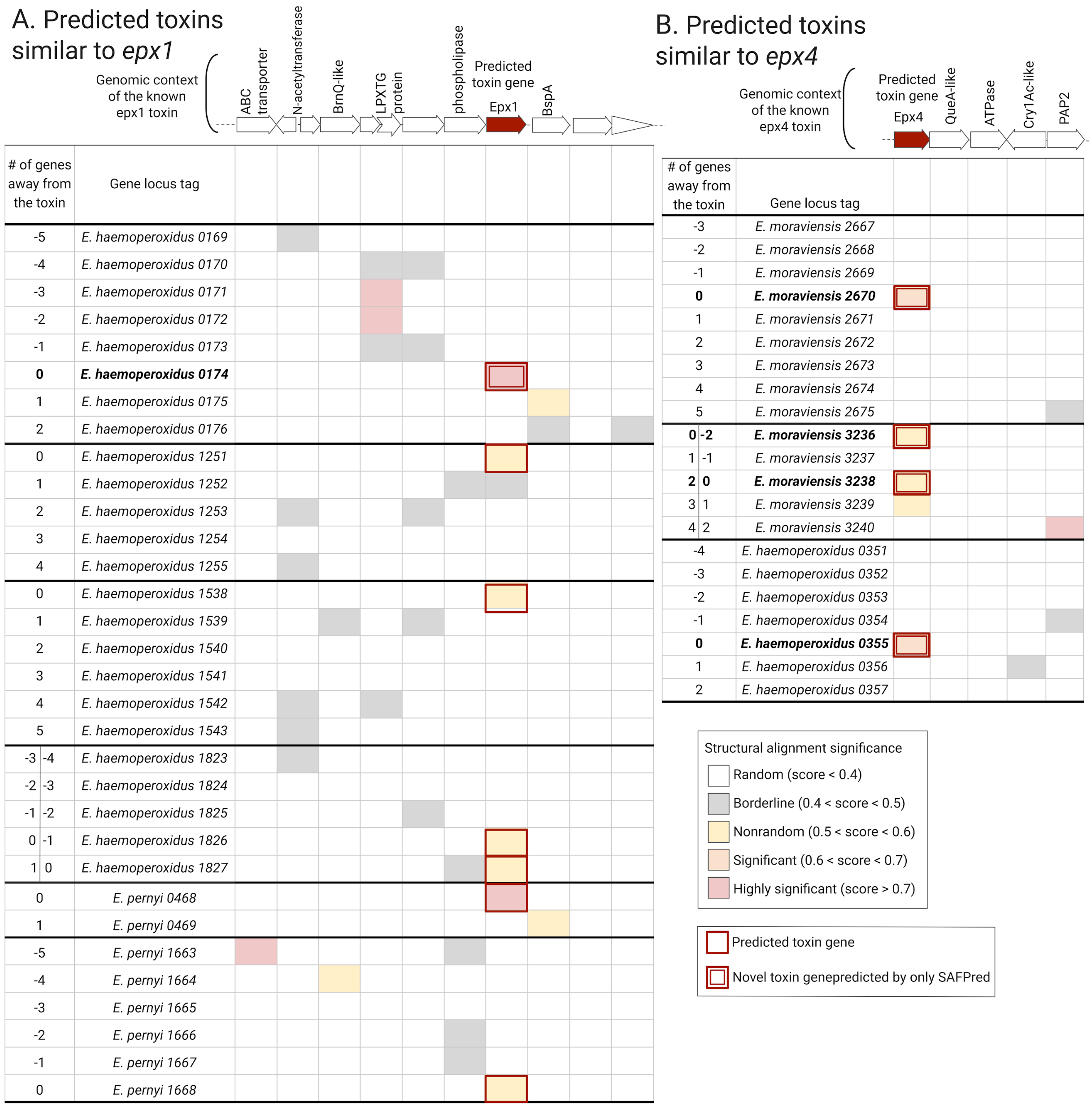
A. Predicted toxins similar to epx1
Genomic context of the known epx1 toxin
ABC transporter
N-acetyltransferase
BrnQ-like
LPXTG protein
phospholipase
Predicted toxin gene
Epx1
BspA
# of genes away from the toxin
Gene locus tag
-5 E. haemoperoxidus 0169
-4 E. haemoperoxidus 0170
-3 E. haemoperoxidus 0171
-2 E. haemoperoxidus 0172
-1 E. haemoperoxidus 0173
0 E. haemoperoxidus 0174
1 E. haemoperoxidus 0175
2 E. haemoperoxidus 0176
0 E. haemoperoxidus 1251
1 E. haemoperoxidus 1252
2 E. haemoperoxidus 1253
3 E. haemoperoxidus 1254
4 E. haemoperoxidus 1255
0 E. haemoperoxidus 1538
1 E. haemoperoxidus 1539
2 E. haemoperoxidus 1540
3 E. haemoperoxidus 1541
4 E. haemoperoxidus 1542
5 E. haemoperoxidus 1543
-3 -4 E. haemoperoxidus 1823
-2 -3 E. haemoperoxidus 1824
-1 -2 E. haemoperoxidus 1825
0 -1 E. haemoperoxidus 1826
1 0 E. haemoperoxidus 1827
0 E. pernyi 0468
1 E. pernyi 0469
-5 E. pernyi 1663
-4 E. pernyi 1664
-3 E. pernyi 1665
-2 E. pernyi 1666
-1 E. pernyi 1667
0 E. pernyi 1668
B. Predicted toxins similar to epx4
Genomic context of the known epx4 toxin
Predicted toxin gene
Epx4
QueA-like
ATPase
Cry1Ac-like
PAP2
# of genes away from the toxin
Gene locus tag
-3 E. moraviensis 2667
-2 E. moraviensis 2668
-1 E. moraviensis 2669
0 E. moraviensis 2670
1 E. moraviensis 2671
2 E. moraviensis 2672
3 E. moraviensis 2673
4 E. moraviensis 2674
5 E. moraviensis 2675
0 -2 E. moraviensis 3236
1 -1 E. moraviensis 3237
2 0 E. moraviensis 3238
3 1 E. moraviensis 3239
4 2 E. moraviensis 3240
-4 E. haemoperoxidus 0351
-3 E. haemoperoxidus 0352
-2 E. haemoperoxidus 0353
-1 E. haemoperoxidus 0354
0 E. haemoperoxidus 0355
1 E. haemoperoxidus 0356
2 E. haemoperoxidus 0357
Structural alignment significance
Random (score < 0.4)
Borderline (0.4 < score < 0.5)
Nonrandom (0.5 < score < 0.6)
Significant (0.6 < score < 0.7)
Highly significant (score > 0.7)
Predicted toxin gene
Novel toxin genepredicted by only SAFPred

**Supplementary Fig. S38.** Detailed view of the genomic neighborhood of 11 likely novel toxin genes (red boxes under *epx* columns). Each row corresponds to a gene within the genomic neighborhood of the 11 predicted toxin genes. Genes are numbered according to their relative position within the operon in reference to the predicted toxin gene (column titled “# of genes away from the predicted toxin”) and the corresponding gene locus tags, a 4-digit number given based on their location within the genome, are also listed in the column titled “Gene locus tag”. A) Comparison to the known *epx1* neighborhood. B) Comparison to the known *epx4* neighborhood. The two operon diagrams at the top (adapted from Xiong et al., 2022) show the known genomic contexts of *epx1* and *exp4*, while the heatmaps below show protein structural alignment significance for proteins within this genomic context.

# References

Lebreton, F. *et al.* (2017). Tracing the enterococci from paleozoic origins to the hospital. *Cell*, **169**(5), 849–861.

Okuda, S. and Yoshizawa, A. C. (2010). Odb: a database for operon organizations, 2011 update. *Nucleic acids research*, **39**(suppl_1), D552–D555.

Parks, D. H. *et al.* (2021). Gtdb: an ongoing census of bacterial and archaeal diversity through a phylogenetically consistent, rank normalized and complete genome-based taxonomy. *Nucleic Acids Research*, **50**(D1), D785–D794.

Seemann, T. (2014). Prokka: rapid prokaryotic genome annotation. *Bioinformatics*, **30**(14), 2068–2069.

Xiong, X. *et al.* (2022). Emerging enterococcus pore-forming toxins with mhc/hla-i as receptors. *Cell*, **185**(7), 1157–1171.e22.

Zhou, N. *et al.* (2019). The CAFA challenge reports improved protein function prediction and new functional annotations for hundreds of genes through experimental screens. *Genome Biology*, **20**(1), 244.
